# Single-cell profiling of human megakaryocyte-erythroid progenitors identifies distinct megakaryocyte and erythroid differentiation pathways

**DOI:** 10.1186/s13059-016-0939-7

**Published:** 2016-05-03

**Authors:** Bethan Psaila, Nikolaos Barkas, Deena Iskander, Anindita Roy, Stacie Anderson, Neil Ashley, Valentina S. Caputo, Jens Lichtenberg, Sandra Loaiza, David M. Bodine, Anastasios Karadimitris, Adam J. Mead, Irene Roberts

**Affiliations:** Centre for Haematology, Department of Medicine, Hammersmith Hospital, Imperial College London, London, UK; Hematopoiesis Section, Genetics and Molecular Biology Branch, National Human Genome Research Institute, National Institutes of Health, Bethesda, MD USA; MRC Molecular Haematology Unit, Weatherall Institute of Molecular Medicine, University of Oxford and BRC Blood Theme, NIHR Oxford Biomedical Centre, Oxford, UK; Department of Paediatrics, Weatherall Institute of Molecular Medicine, University of Oxford and BRC Blood Theme, NIHR Oxford Biomedical Centre, Oxford, OX3 9DU UK; Flow Cytometry Core, Genetics and Molecular Biology Branch, National Human Genome Research Institute, National Institutes of Health, Bethesda, MD USA

**Keywords:** Hematopoiesis, megakaryopoiesis, erythropoiesis, thrombopoiesis, myelopoiesis, hematopoietic stem cell

## Abstract

**Background:**

Recent advances in single-cell techniques have provided the opportunity to finely dissect cellular heterogeneity within populations previously defined by “bulk” assays and to uncover rare cell types. In human hematopoiesis, megakaryocytes and erythroid cells differentiate from a shared precursor, the megakaryocyte-erythroid progenitor (MEP), which remains poorly defined.

**Results:**

To clarify the cellular pathway in erythro-megakaryocyte differentiation, we correlate the surface immunophenotype, transcriptional profile, and differentiation potential of individual MEP cells. Highly purified, single MEP cells were analyzed using index fluorescence-activated cell sorting and parallel targeted transcriptional profiling of the same cells was performed using a specifically designed panel of genes. Differentiation potential was tested in novel, single-cell differentiation assays. Our results demonstrate that immunophenotypic MEP comprise three distinct subpopulations: “Pre-MEP,” enriched for erythroid/megakaryocyte progenitors but with residual myeloid differentiation capacity; “E-MEP,” strongly biased towards erythroid differentiation; and “MK-MEP,” a previously undescribed, rare population of cells that are bipotent but primarily generate megakaryocytic progeny. Therefore, conventionally defined MEP are a mixed population, as a minority give rise to mixed-lineage colonies while the majority of cells are transcriptionally primed to generate exclusively single-lineage output.

**Conclusions:**

Our study clarifies the cellular hierarchy in human megakaryocyte/erythroid lineage commitment and highlights the importance of using a combination of single-cell approaches to dissect cellular heterogeneity and identify rare cell types within a population. We present a novel immunophenotyping strategy that enables the prospective identification of specific intermediate progenitor populations in erythro-megakaryopoiesis, allowing for in-depth study of disorders including inherited cytopenias, myeloproliferative disorders, and erythromegakaryocytic leukemias.

**Electronic supplementary material:**

The online version of this article (doi:10.1186/s13059-016-0939-7) contains supplementary material, which is available to authorized users.

## Background

Hematopoietic stem cells (HSC) give rise to blood cells of multiple lineages through step-wise lineage restriction and the production of intermediate oligo- and bipotent progenitors. In the traditional hierarchical model, HSC sequentially differentiate into multipotent progenitors (MPP) then common lymphoid progenitors (CLP) and myeloid progenitors (CMP), the latter of which give rise to granulocyte-macrophage progenitors (GMP) and megakaryocyte-erythroid progenitors (MEP), which in turn bifurcate to lineage-committed erythroid cells and platelet-producing megakaryocytes [1, 2]. The cellular populations in the HSC/progenitor hierarchy are distinguished by antibodies that identify differential expression of cell surface antigens. However, the molecular and cell biology studies that defined these populations have largely been carried out in “bulk” assays, which will fail to detect cellular subfractions. Indeed, recent studies of human hematopoiesis have questioned this classical view of lineage development, raising the possibility that human hematopoietic progenitor populations are composed of heterogeneous and lineage-restricted subpopulations [3].

Advances in single-cell techniques, in particular transcriptional profiling and associated computational strategies, now allow for a more complete investigation of the cellular heterogeneity that may exist within populations [4–7]. For example, in the murine hematopoietic system, single-cell approaches have identified cells transcriptionally primed towards different fates within the myeloid progenitor population while rare, megakaryocyte-primed multipotent progenitor cells that arise directly from HSC and can bypass the MEP in stress or emergency megakaryopoiesis have been demonstrated both in mice and zebrafish [8–12]. These studies demonstrate the power of single-cell gene expression techniques to challenge the conventional models of hematopoiesis by uncovering heterogeneity within phenotypically defined cell populations.

Accordingly, a better understanding of the cellular hierarchy underlying the differentiation of bipotent MEP to erythroid and megakaryocyte lineage-committed progenitors (EP/MKP) is required. Although several immunophenotyping approaches have been proposed to enrich for human MEP [13–15], under the two best validated strategies, MEP are negatively defined and distinguished from CMP and GMP by the absence of the surface antigens CD123 [13] or Flt3/CD135 [15]. This results in a heterogeneous population enriched for lineage-biased or committed megakaryocyte and erythroid progenitors/precursors with variable contamination with myeloid progenitors [14, 16–18]. Consistent with this, erythroid-primed MEP and committed EP were recently identified within the immunophenotypic MEP compartment by their differential expression of the surface antigens CD71 and CD105 [17, 18]. This raises the possibility that analogous megakaryocyte-primed MEP and MKP may also exist within this population, similar to those characterized in the murine system [19, 20], although this has yet to be demonstrated.

**Fig. 1:**
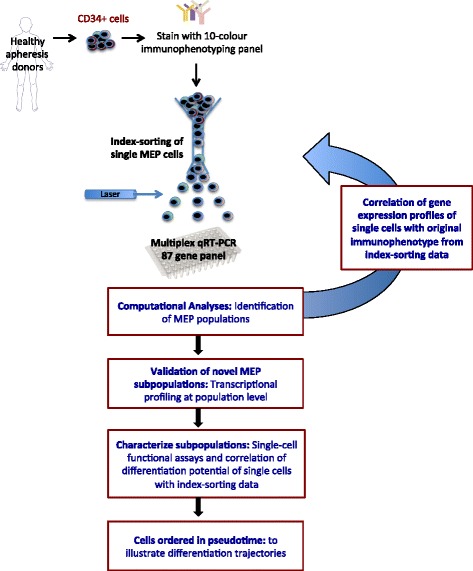
Overview of the experimental strategy. CD34+ cells from healthy, mobilized apheresis donors were immunostained with a 10-fluorochrome panel and single cells were index-sorted into 96-well PCR plates for multiplex qRT-PCR analysis using the Fluidigm Biomark platform. MEP subpopulations were identified by principal component analysis (PCA) and correlated with the original index sorting data and mRNA levels of surface antigens. Identified cellular subsets were validated transcriptionally at the population level and functionally in single-cell differentiation assays. Finally, the cells were ordered in pseudotime to assess differentiation trajectories which were then further validated in functional assays. FACS, fluorescence-activated cell sorting; IF, immunofluorescence; qRT-PCR, quantitative real-time polymerase chain reaction

The ability to map transcriptional and functional profiles with cell surface protein expression at the single-cell level enables a more detailed examination of the homogeneity of a population than was previously possible [21–23]. In this report, we illustrate the power of this approach by applying combined transcriptional, phenotypic, and functional single-cell analyses to detect and validate novel subpopulations within classically defined, immunophenotypic lineage (Lin)- CD34 + CD38 + CD123- CD45RA- MEP (Fig. 1). We first performed targeted expression profiling of 87 genes in 681 single human MEP cells isolated using index fluorescence-activated cell sorting (FACS). Integrating individual cell surface immunophenotype and transcriptional profiles with functional output in novel, single-cell differentiation assays revealed that the Lin- CD34 + CD38 + CD123- CD45RA- “MEP” population in fact comprises three distinct subpopulations: (1) “Pre-MEP/CMP,” enriched for bipotent erythroid/megakaryocyte progenitors with residual myeloid differentiation capacity; (2) “E-MEP,” which are strongly biased towards erythroid differentiation; and (3) “MK-MEP,” a less frequent population of bipotent cells that primarily generate megakaryocytic progeny. Our study elucidates a novel cellular hierarchy in megakaryocyte/erythroid lineage commitment and an immunophenotyping strategy to enable prospective identification of specific populations, thereby enabling in-depth study of clinically important disorders of erythro-megakaryopoiesis, including inherited cytopenias, myeloproliferative disorders, and erythromegakaryocytic leukemias.

## Results and discussion

### Single-cell gene expression analysis reveals heterogeneity within phenotypically defined human MEP

**Fig. 2:**
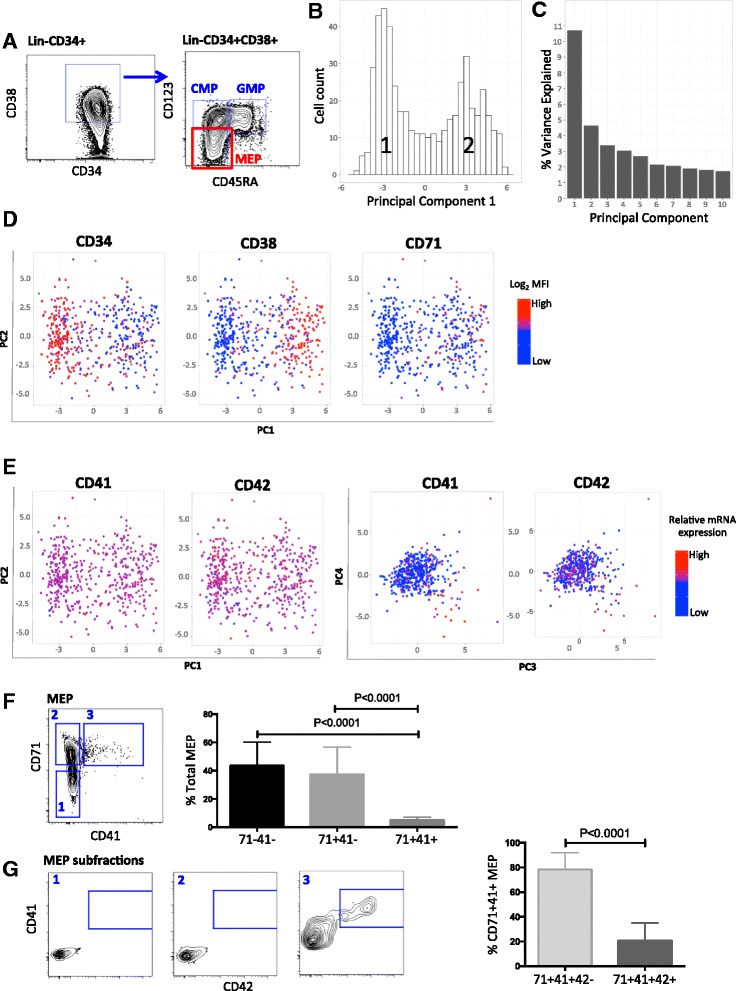
Single-cell gene expression analysis demonstrates significant cellular heterogeneity and the presence of subpopulations within classically defined, immunophenotypic MEP. **a** A previously validated strategy was used to distinguish MEP from the other lineage-negative (Lin-) CD34+ CD38+ myeloid progenitor populations—common myeloid (CMP) and granulocyte-macrophage progenitors (GMP)—by the absence of CD123 and CD45RA. Quantification gates are shown (sorting gates are shown in Additional file 1: Figure S1A). **b** Multiplex qPCR of 87 genes in 489 Lin- CD34 + CD38 + CD123- CD45RA-MEP cells and PCA was performed. The distribution of cells along PC 1 demonstrates two distinct cellular populations (annotated 1 and 2). **c** *Plot* showing % variance by PCs 1–10. **d** Superimposition of mean log_2_ fluorescence intensity (MFI) values of the original cells isolated for qPCR on the PCA for PC1 and PC2 reveals that the two populations have distinct expression profiles for CD34, CD38, and CD71. **e** Superimposition of CD41 and CD42 expression on the PCA for PC1 vs*.* PC2 (MFI, *left plots*) indicated rare cells with high CD41 and CD42 expression which did not fall into either Population 1 or 2, suggesting the presence of smaller subpopulation(s) expressing megakaryocyte-associated antigens. CD41^high^ and CD42^high^ cells segregated more distinctly by PC3 vs*.* PC4 (relative mRNA expression, *right plots*). *Red-blue scale* indicates high to low expression (customized for each plot in 2D and 2E). **f** Representative *flow plot* (*left*) illustrating differential expression of CD71 and CD41 within immunophenotypic MEP compartment, identifying three subpopulations: (1) CD71-CD41-; (2) CD71 + 41- ; and (3) CD71 + 41+. Quantification of these three subpopulations (*right*) in CD34+ cells from 14 healthy donors. Cells falling between FACS gates are excluded from the chart. CD71 + 41 + MEP are significantly less frequent, constituting 5.1 ± 0.6 % of total MEP (mean ± SEM, *P* <0.0001). **g** Expression of CD42 in the three MEP subfractions. CD42 expression is restricted to a minority (20.7 ± 4.1 %) of CD71 + CD41 + MEP cells (*P* <0.0001)

To isolate MEP, we adapted a previously validated immunophenotyping strategy in which MEP are distinguished from the other lineage-negative (Lin-) CD34 + CD38+ hematopoietic progenitors, CMP and GMP, by the absence of CD123 and CD45RA (Fig. 2a, Additional file 1: Figure S1A) [24]. To test the hypothesis that cellular heterogeneity exists within the MEP compartment, including cells primed for megakaryocyte versus erythroid differentiation, we analyzed 489 Lin- CD34 + CD38 + CD123- CD45RA- human MEP cells from three healthy donors. Individual cells were isolated by index FACS sorting using a panel of nine cell surface markers (Additional file 1: Figure S1A). Single-cell gene expression profiling was performed by multiplex RT-PCR using a customized panel of 87 genes, enabling correlation of individual cell surface immunophenotype and gene expression profiles. This gene set included genes predicted to be differentially regulated during erythroid and megakaryocyte differentiation according to published RNA-Seq datasets from bulk-sorted, human MEP and mature erythroid and megakaryocyte populations [25]; cell surface antigens known to be expressed during erythroid and megakaryocytic differentiation [8, 25]; and three housekeeping genes. Principal component analysis (PCA) revealed that MEP were clearly segregated into two distinct subpopulations by principal component (PC) 1 (Fig. 2b), which accounted for 10.72 % of the variance in gene expression between cells (Fig. 2c and Additional file 1: Figure S1B). No important plate or sample effect was observed (Additional file 1: Figure S1C–F).

CD71 and CD41 are early identifiers of erythroid and megakaryocyte progenitors, respectively [17, 18, 26]. CD42 (glycoprotein 1b) is expressed later during megakaryocyte differentiation and has been associated with unipotent megakaryopoietic activity in mouse models [26]. These antigens were therefore included in the immunophenotyping panel used to isolate the original cells for gene expression profiling and the intensity of surface expression (mean fluorescence intensity [MFI]) was superimposed on the PCA. This indicated that the two cellular subsets identified by PCA (Population 1 and 2) were distinguishable by their surface expression of CD34, CD38, and CD71 (Fig. 2d). Population 1 (left) contained cells with higher CD34 and lower CD38 expression, suggesting a more immature phenotype (Fig. 2d), while Population 2 (right) contained cells with higher CD71 expression (Fig. 2d). Infrequent cells with distinctly higher expression of CD41 and CD42 were notable which did not clearly cluster with either population by PC1 (Fig. 2e) although the CD41-high cells separated more distinctly in PCs 3 and 4 (Fig. 2e). We reasoned that these cells might represent megakaryocyte-primed MEP that do not form a separate cluster on the PCA by PC1 due to their relatively low frequency.

We next directly analyzed the cell surface expression of CD71, CD41, and CD42 within Lin- CD34 + CD38 + CD123- CD45RA- MEP of peripheral blood CD34+ cells from 14 healthy, G-CSF-treated donors (Fig. 2f, g). In keeping with the PCA, two subpopulations could be distinguished by their differential expression of CD71 and a third by the expression of CD41: (1) CD71-41- (43.6 ± 4.8 % of total MEP); (2) CD71 + 41- (37.4 ± 3.6 %); and (3) CD71 + 41+, which was significantly less frequent than the other two populations (5.1 ± 0.6 %, Fig. 2f, *P* <0.0001). CD42 expression was restricted to ~1/5 of CD71 + 41 + MEP cells, or ~1 % of total MEP (Fig. 2g).

**Fig. 3:**
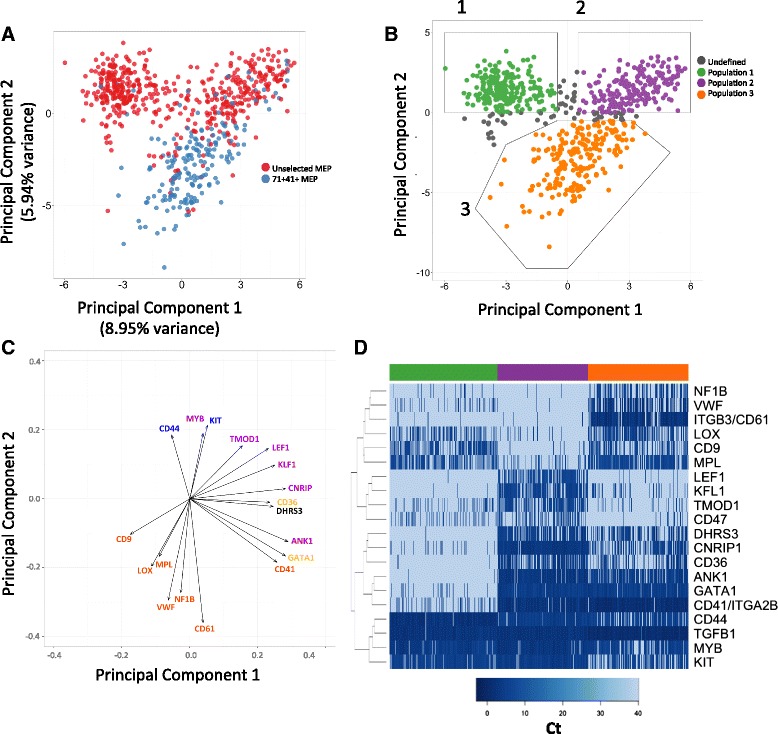
MEP contain three distinct subpopulations segregated by differential expression of megakaryocyte and erythroid-associated genes. **a** PCA of 681 cells showing distribution of unselected MEP cells (n = 489; *red*) and CD71 + 41+ selected MEP (n = 192; *blue*) for PC 1 (8.95 % variance) and PC2 (5.94 % variance). CD71 + 41+ MEP are distinct from Populations 1 and 2. **b** The three subpopulations that emerged from the PCA (Fig. 2a) were defined as Populations 1 (*green*), 2 (*purple*), and 3 (*orange*) on the basis of PC1 and PC2 values. **c** The 18 most highly weighted genes in PC1 and 2 show that the distinction of the populations is driven by differential expression of key megakaryocyte (*orange font*) and erythroid-associated (*purple font*) genes. *Blue font* indicates genes associated with more primitive cellular phenotype (*CD44* and *KIT*). *Black* indicates an MEP gene (*DHRS3*) and *yellow* (*GATA1*, *CD36*) genes expressed in both megakaryocytic and erythroid cells. **d** *Heatmap* of Ct values shows differential gene expression of 20 selected genes between the three populations identified on the PCA. (*Green*, Population 1; *purple*, Population 2; *orange*, Population 3)

We then explored the possibility that the CD71 + 41- and CD71 + 41 + MEP subfractions might represent erythroid and megakaryocyte-primed populations, respectively. Due to the rarity of the CD71 + 41+ MEP cells, we selectively analyzed an additional 192 CD71 + CD41+ MEP cells from the three same donors by index-FACS sorting for gene expression profiling. When all 681 analyzable cells (489 unselected MEP plus 192 71 + 41+ MEP) were studied, PCA demonstrated that 71 + 41+ MEP constituted a distinct third population (Fig. 3a), allowing us to identify three distinct populations on the basis of PCs 1 and 2 for each individual cell (Fig. 3b). Cells expressing highest levels of surface CD42 by FACS appeared at the apex of Population 3 in the PCA (Additional file 1: Figure S2A).

Depicting the data by non-linear dimensionality reduction (t-distributed stochastic neighbor embedding, t-SNE) analysis [27–30] also demonstrated three subpopulations, supporting the PCA (Additional file 1: Figure S2B). To determine whether gene choice was a strong determinant of the three subpopulation substructure apparent on PCA and t-SNE, random subsets of genes were selected to perform PCA and the proportion of cells that were congruently assigned to each original population was ascertained (Additional file 1: Figure S2C). This demonstrated that on average 75 % of the cells are assigned equivalently with as few as 25 genes. Furthermore, to confirm that the PCA was not substantially biased by drop-out events, Zero Inflated Factor Analysis (ZIFA) was performed (Additional file 1: Figure S2D) [31]. In accordance with PCA and t-SNE, ZIFA also segregated the MEP cells into three populations (Additional file 1: Figure S2D).

Identifying the 18 most highly weighted genes in PC1 and PC2 (Fig. 3c) and the heatmap of gene expression (Fig. 3d, Additional file 1: Figure S2E) revealed that the segregation of the three populations was driven by differential expression of megakaryocyte-associated and erythroid- associated genes. Hierarchical clustering of the gene expression profiles also supported the division of Lin- CD34 + CD38 + CD123- CD45RA- MEP into three subpopulations (Additional file 1: Figure S2F).

### Three MEP subpopulations can be prospectively identified immunophenotypically by their differential expression of CD44, CD71, and CD41

**Fig. 4:**
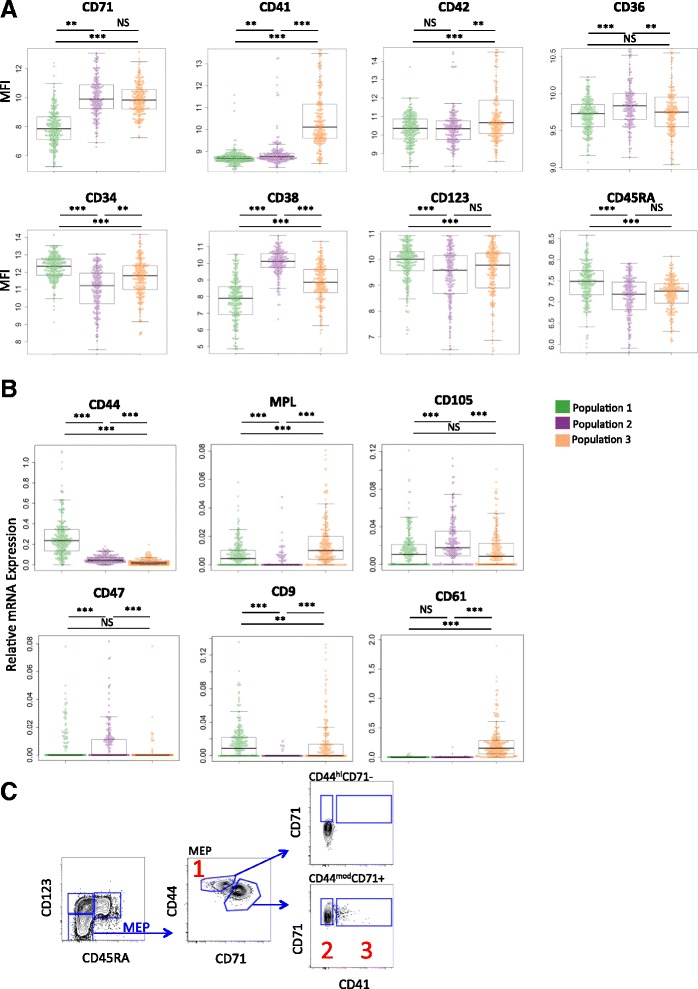
Cell surface antigen expression discriminates the three MEP subpopulations identified by single-cell gene expression analysis. **a** Mean fluorescence intensity (MFI) of eight surface antigens included in the FACS panel for the three populations assigned by PCA. Population 1 (*green*) contained cells with significantly higher CD34, CD123, and CD45RA and lowest CD38, CD71, CD41, and CD42 expression. Population 2 (*purple*) identified as CD71 + 41- and Population 3 (*orange*) as CD71 + 41+. **b** Cell surface antigens included in the qPCR profile panel but not the FACS panel were considered to further refine the immunophenotyping strategy. CD44 expression emerged from the qPCR data as the most differentially expressed surface antigen associated with Population 1 (*P* <0.0001). *Star indicators* represent significance values (KS test with FDR correction) between populations: *-q <0.05; **-q <0.01; ***-q <0.001; NS-q >0.05. Data are shown as *bee-swarm plots* in which the log_2_ MFI values (**a**) or relative mRNA expression level (b) of individual cells are represented as *dots* with a *box plot* overlaid. **c** The utility of CD44 immunophenotyping was validated by flow cytometry, confirming that high surface expression of CD44 correlates with the CD71- CD41- MEP subfraction. *Numbers* shown correspond to the three MEP subsets: Population 1, CD44^hi^ 71- 41- ; population 2, CD71 + 41-; population 3, CD71 + 41+

To determine whether FACS could be used to identify the three MEP subpopulations that emerged from PCA of gene expression, we next determined the mean fluorescence intensity of antigens in our FACS panel for the original cells index-sorted for gene expression profiling (Fig. 4a). The three subpopulations of MEP identified by PCA could be distinguished with high sensitivity and specificity (specificity range of 0.81–0.91; sensitivity 0.67–0.90; Additional file 1: Figure S3A) using a combination of CD71 and CD41: (1) CD71-41-; (2) CD71 + 41-; and (3) CD71 + 41+. Further, although all of the single MEP cells had been sorted from the Lin- CD34 + CD38 + CD123- MEP gate (Fig. 2a, Additional file 1: Figure S1A), CD71-41- MEP (Population 1) had relatively higher CD34, lower CD38, and higher CD123 and CD45RA surface antigen expression (Fig. 4a), suggesting they might be positioned earlier in the HSC/progenitor hierarchy. Expression of the early erythroid/megakaryocyte marker CD36 was lowest in Populations 1 and 3 but did not discriminate clearly between the MEP populations, and CD42 expression was highest in Population 3 (Fig. 4a). The cell surface phenotypes showed highly significant correlation with mRNA levels of the same surface proteins (Additional file 1: Figure S3B). Taken together, these data indicate that Lin- CD34 + CD38 + CD123- CD45RA- MEP constitute a heterogeneous population of cells with at least three distinct subpopulations that can be distinguished by unique surface marker and transcript profiles.

Because Population 1 remained negatively defined among CD34 + 38+ hematopoietic progenitors (Lin- CD34 + CD38 + CD123- CD45RA-CD71- CD41-), we sought to determine whether our immunophenotyping strategy for this population could be further refined by including additional surface antigens from our gene expression profiling panel that were not part of the original FACS panel. CD44, an adhesion molecule expressed by MEP and early erythroid and megakaryocytic progenitors that is downregulated during their differentiation and maturation [32, 33] emerged as the most prominent positive identifier of Population 1 by gene expression, with a mean expression level fivefold higher than the other two populations (*P* <0.0001, Fig. 4b). Other erythroid/megakaryocytic surface antigen genes were either barely expressed in Population 1 (*CD61*) or were expressed at similar levels in Populations 1 and 3 (*CD9*) or in all three populations (*CD105*, *CD47*) (Fig. 4b). *MPL* expression was detectable in all three MEP subpopulations, in keeping with previous reports [14], indicating that MPL is unlikely to be a good candidate marker to differentiate between the three populations by immunophenotyping (Fig. 4b).

To confirm the utility of CD44 as a positive identifier of this population by immunophenotyping, CD44 was incorporated into our 10-fluorochrome panel. This allowed us to separate the MEP population immunophenotypically into CD44^hi^CD71- CD41- MEP (Fig. 4c), which had similar surface CD44 expression to CMP and GMP (Additional file 1: Figure S3C), and CD44^mod^CD71+ MEP, which contained all of the CD71 + 41- and CD41+ MEP cells (Fig. 4c). These data confirmed that the differential expression patterns of CD44, CD71, and CD41 enable positive identification and prospective isolation of all three MEP subpopulations. To confirm that the addition of CD44 to the immunophenotyping panel defined the transcriptome-identified subpopulations, 100 cells were sorted from each of the three MEP populations as defined by CD44, CD71, and CD41 co-expression as shown in Fig. 4c, in triplicate from each of four donors. Multiplex RT-PCR analysis performed using the same panel of gene expression assays used for the single-cell transcriptional profiling confirmed that the cells purified according to this novel surface phenotype strategy also showed transcriptional profiles as seen in the original single-cell analyses (Additional file 1: Figure S3D and 3E).

### Differential expression of key megakaryocyte and erythroid genes between the MEP subpopulations indicates a “Pre-MEP,” “E-MEP,” and “MK-MEP” transcriptional profile

**Fig. 5:**
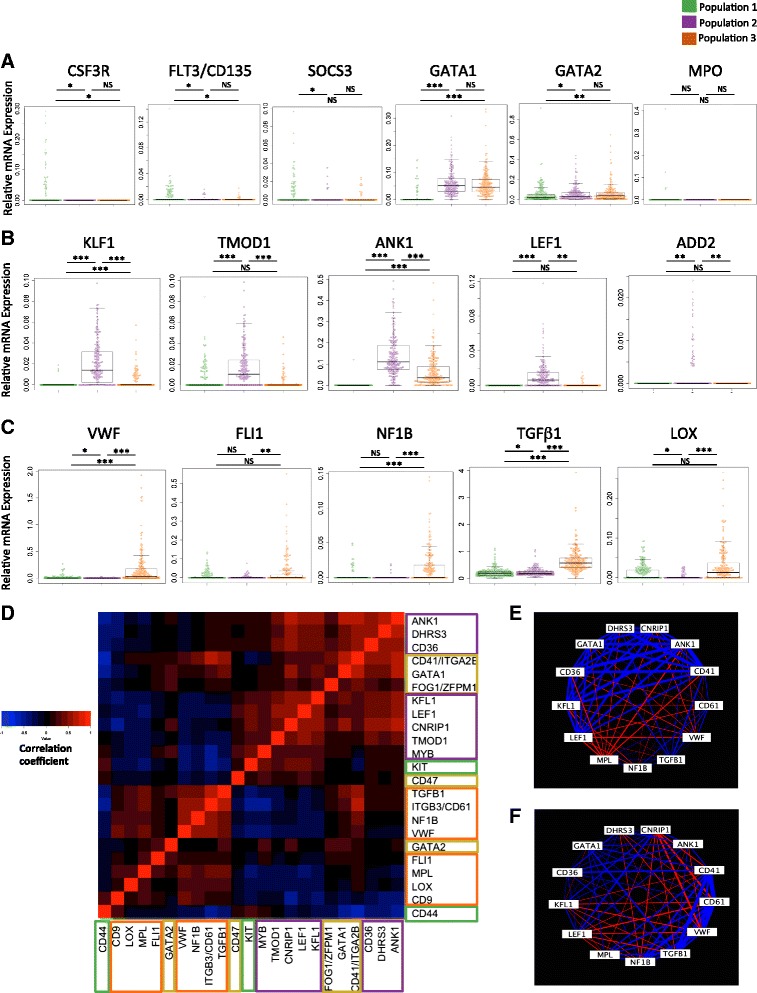
Distinct erythroid-associated and megakaryocyte-associated transcriptional lineage-priming in MEP subpopulations. **a** Population 1 (*green*) contained cells with residual *CSF3R*, *FLT3/CD135*, and *SOCS3* expression and lowest *GATA1* and *GATA2* expression, suggesting that this population comprises progenitors earlier in the hematopoietic hierarchy than populations 2 and 3 and more closely related to CMP. Expression of myeloperoxidase (*MPO*) was only detected in five of 681 cells, indicating minimal contamination of the FACS-isolated MEP cells with CMP or other myeloid lineage cells, in which MPO is strongly positive [20]. **b** The highest levels of expression of erythroid genes, including *KLF1*, *TMOD1*, *ANK1*, *LEF1*, and *ADD2* were observed in Population 2 (*purple*). **c** The highest levels of expression of megakaryocyte genes, including *VWF*, *FLI1*, *NFIB*, *TGFB1*, and *LOX* occurred in Population 3 (*orange*). Each chart shows a *bee-swarm plot* where each *dot* represents the gene expression of an individual cell, with a *box plot* overlaid. Significance values are shown for q-values for KS test with FDR correction between populations: *-q <0.05; **-q <0.01; ***-q <0.001; NS-q >0.05. **d** *Heatmap* showing correlation of expression of selected erythroid and megakaryocytic genes within single cells. Color-coding: *Orange box*, megakaryocyte gene set; *purple*, erythroid; *yellow*, both megakaryocyte and erythroid; *green*, genes associated with pre-MEP phenotype. **e**, **f** Representation of Spearman correlation coefficient between selected genes in populations 2 (Fig. 5e) and 3 (Fig. 5f), respectively. *Blue edges* denote positive correlation and *red edges* denote negative correlation. *Edge thickness* is a function of correlation magnitude

Significant differences were observed between these three populations in the expression of key erythroid and megakaryocyte genes (Fig. 5a–c). A higher proportion of cells in Population 1 expressed *CSF3R* (the granulocyte-colony stimulating factor [G-CSF] receptor), *FLT3/CD135*, and *SOCS3* than Populations 2 and 3 and expression of the key erythroid-megakaryocytic transcription factors *GATA1* and *GATA2* were significantly lower in this population (Fig. 5a) consistent with a less differentiated state. Expression of myeloperoxidase (*MPO*), a gene abundantly expressed by granulocytes, CMP and GMP [34], was undetectable in all but five of 681 cells in all three populations (Fig. 5a), confirming that contamination of the sorted populations with CMP or other myeloid cells/progenitors was negligible. Expression of genes encoding erythroid transcription factors and membrane proteins, e.g. *KLF1*, *CD71*, *TMOD1*, *ANK1*, and *LEF1* was significantly higher in Population 2 (Figs. 3d and 5b), while Population 3 showed highest expression of megakaryocyte-associated proteins, including *VWF*, *FLI1*, *NFIB*, *TGFβ*, and *LOX* (Figs. 3d and 5c). Correlations of megakaryocytic (*CD9*, *LOX*, *MPL*, *VWF*, *NFIB*, *CD61*, *TGFβ*, *FLI1*) and erythroid (*CD36*, *KLF1*, *LEF1*, *CNRIP1*, *TMOD1*, *MYB*) gene sets and megakaryocyte-erythroid transcription factors (*GATA1*, *GATA2*, *FOG1*) in all cells suggested co-regulation of same-lineage and repression of alternate-lineage pathways (Fig. 5d). Moreover, we also found distinct erythroid and megakaryocytic gene co-expression patterns (within the same single cells) in Population 2 and 3, respectively (Fig. 5e and 5f). On the basis of these data, we defined Population 1 as “pre-MEP/CMP-like” (“Pre-MEP”), Population 2 as erythroid-primed MEP (“E-MEP”), and Population 3 as megakaryocyte-primed MEP (“MK-MEP”).

### Single-cell differentiation assays demonstrate that the lineage bias suggested by transcriptional and cell surface profiles correspond to functional differences in lineage differentiation

**Fig. 6:**
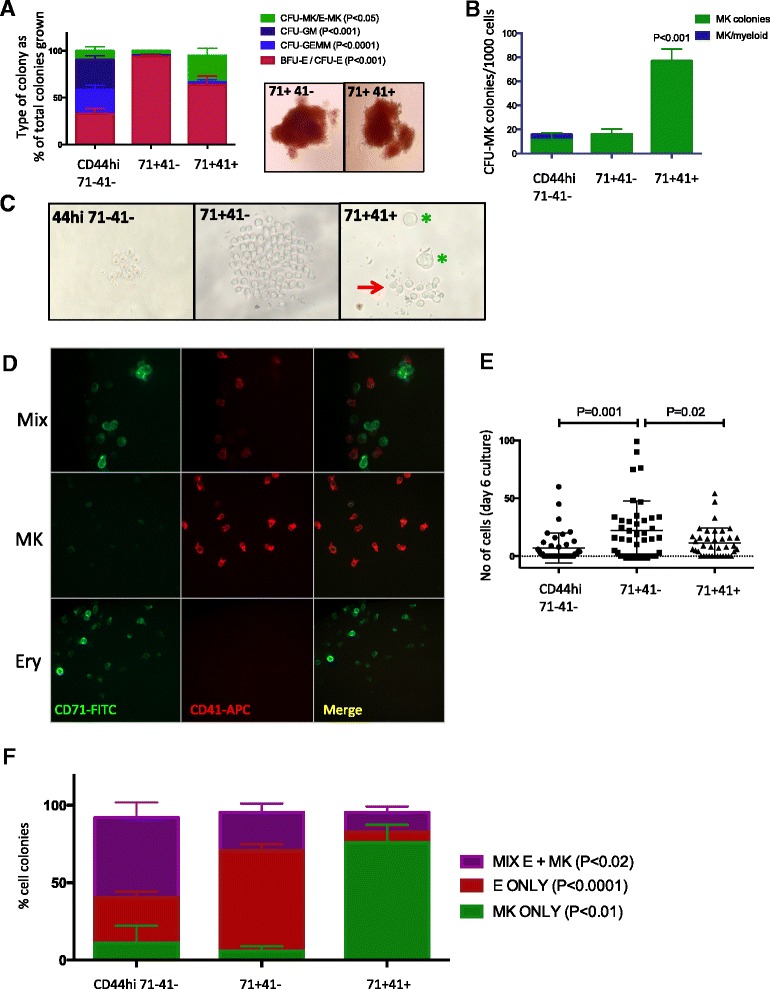
Single-cell functional assays confirm erythroid and megakaryocyte differentiation bias of CD71 + 41- MEP and CD71 + 41+ MEP, whereas CD44^hi^71- 41- MEP demonstrate a “Pre-MEP” phenotype. **a** Colony-forming capacity of single MEP cells in methylcellulose, which primarily supports erythroid and myeloid differentiation. *Left graph*: colony phenotype as a percentage of total colonies grown. The percentage of erythroid colonies (BFU-E/CFU-E; *red*) was significantly higher for CD71 + 41- MEP than the other two populations. CD44^hi^CD71-41- MEP cells generated a higher percentage of myeloid colonies (CFU-GEMM/GM; *blue*) than the CD71+ fractions. Photographs show representative BFU-Es derived from single CD71 + 41- and CD71 + 41+ cells. Data shown are for 30–60 single cells sorted from each population in each of seven separate experiments. **b** Megakaryocyte colony-forming potential was tested in a collagen-based assay supporting megakaryocyte and myeloid but not erythroid colonies. CD71 + 41+ MEP cells gave rise to significantly more megakaryocyte colonies (*green*; n = 4). **c** Typical cell cultures 6 days after seeding of single cells from MEP subsets into a liquid culture system supporting erythroid and megakaryocytic differentiation. An example of a mixed megakaryocyte and erythroid colony is shown for CD71 + 41+ MEP, with two large, proplatelet-forming megakaryocytes (*green stars*) and several smaller erythroblasts (*red arrow*). The example colonies shown for CD44^hi^CD71- CD41- and CD71 + CD41- MEP are exclusively erythroid, with a higher proliferation rate in the CD71 + 41- colony. **d** The identity of cells in individual culture wells was determined by immunofluorescence (IF) microscopy, flow cytometry, and morphology. Example IF images of mixed (Mix, *top*), megakaryocyte-only (MK, *middle*), and erythroid-only (Ery, *bottom*) cultures. Cells immunostained for CD71 (FITC, *green*) and CD41(APC, *red*). **e** Cell number in each well 6 days after seeding with a single cell. CD71 + 41- MEP are most proliferative. N = 3. **f** Summary FACS data for 100 single-cell colonies analyzed (n = 3). CD44^hi^CD71- 41-MEP most frequently generated mixed erythroid/megakaryocyte colonies; CD71 + 41- showed mostly erythroid-only and CD71 + 41+ showed primarily MK-only progeny. *P* values are for one-way ANOVA between populations

To validate that the lineage bias suggested by transcriptional and cell surface profiles corresponded to functional differences in the ability of the cells to differentiate, we analyzed Pre-MEP, E-MEP, and MK-MEP in single-cell differentiation assays. Single cells from the three MEP populations were seeded by FACS according to the strategy shown in Fig. 4c into conventional colony-forming assays in semi-solid medium. Erythroid and myeloid colony-forming capacity was tested in methylcellulose assays, which support the growth of erythroid, myeloid, and to a lesser extent megakaryocytic colonies. Colonies were classified as myeloid or erythroid by visual inspection (Fig. 6a); indeterminate colonies were plucked for analysis of lineage-associated surface antigens by flow cytometry. There was a marked difference in colony output from the three MEP populations that matched their transcriptional profile (Fig. 6a). Over 90 % of colonies arising from single CD71 + 41- E-MEP cells were erythroid (BFU-E/CFU-E), compared to ~60 % of colonies arising from single CD71 + 41+ cells and ~30 % of CD44^hi^71- 41- MEP colonies (*P* <0.001, Fig. 6a). Wells seeded with CD71 + 41- E-MEP also had the highest colony-forming efficiency overall, with colonies detected in almost 60 % of wells, as compared to ~40 % of wells seeded with CD44^hi^CD71- 41-Pre-MEP and ~20 % of wells seeded with CD71 + 41 + MK-MEP (Additional file 1: Figure S4A). Myeloid colonies were very rarely observed in wells seeded with E-MEP and MK-MEP cells, while mixed granulocyte-erythroid-macrophage-megakaryocyte colonies (CFU-GEMM) and pure myeloid (granulocyte-macrophage, CFU-GM) colonies each constituted 25–30 % of total colonies derived from Pre-MEP (Fig. 6a). This demonstrated that E-MEP and MK-MEP were almost exclusively committed to erythroid-megakaryocytic differentiation. In contrast, Pre-MEP had maintained potential to generate myeloid colonies. Further, Pre-MEP are functionally distinct from CMP being markedly enriched for erythro/megakaryopoietic efficiency as compared to CMP (Additional file 1: Figure S4B), and almost all of the myeloid clonogenic output observed in unfractionated MEP is contained within this fraction. Surface CD44 expression of cells giving rise to myeloid colonies was significantly higher than those giving rise to erythroid colonies, confirming the utility of CD44 as a positive identifier of cells with a Pre-MEP phenotype (Additional file 1: Figure S4C). In contrast, there was no difference in CD123 expression between cells which gave rise to myeloid colonies versus those that gave rise to pure erythroid or erythroid/MK colonies (Additional file 1: Figure S4C).

To test megakaryocyte colony-forming potential, cells from the three MEP populations were sorted into a collagen-based medium that supports megakaryocyte and myeloid colonies (Megacult™). We observed that MK-MEP cells gave rise to fourfold more megakaryocyte colonies than the other subpopulations, in keeping with a megakaryocytic differentiation bias (*P* <0.001, Fig. 6b).

In semi-solid assays, the growth of either myeloid and erythroid colonies (methylcellulose) or myeloid and megakaryocyte colonies (Megacult™) is efficient, but mixed megakaryocyte-erythroid colonies are infrequent and single-cell megakaryocyte colony-forming assays are not possible due to low clonogenic efficiency. Therefore, to identify bipotent cells with the potential to differentiate into both erythroid and megakaryocytic cells, we utilized a specifically designed single-cell liquid culture system optimized to support differentiation of erythroid cells and megakaryocytes. Cells from each MEP fraction were individually seeded into each well of 96-well plates containing medium supplemented with the cytokines required for both erythroid and megakaryocytic differentiation (EPO, TPO, IL3, IL6, SCF) [35, 36]. Wells were inspected 6 days following seeding by light microscopy for the presence of characteristic erythroblasts and proplatelet-forming megakaryocytes (Fig. 6c). The cellular phenotype of the progeny derived from the single cells was identified by morphology and the expression of lineage markers as assessed by fluorescence microscopy and flow cytometry allowing us to identify megakaryocyte-only, erythroid-only, and mixed progeny (Figs. 6d, Additional file 1: Figure S4D).

We used this approach to analyze the three MEP subpopulations. In this liquid culture system, single E-MEP cells were significantly more proliferative than the other two MEP fractions, generating higher numbers of cells 6 days after seeding (Fig. 6e) and had the highest frequency of cells giving rise to populations of exclusively erythroid progeny (Fig. 6f). The highest frequency of pure populations of megakaryocytic cells occurred in cells seeded with the MK-MEP (Fig. 6f). Only a minority of single E-MEP and MK-MEP cells gave rise to “mixed” colonies containing both erythroid and megakaryocytic cells (Fig. 6f). By contrast, mixed colonies occurred in almost 50 % of wells seeded with cells from the Pre-MEP fraction (*P* <0.02, Fig. 6f), which was also able to give rise to both unipotent erythroid and unipotent megakaryocytic cells. Together, these functional data are consistent with our conclusions from the transcriptional profiles and support a definition of the cellular subfractions as: Pre-MEP (CD44^hi^71- 41-); E-MEP (CD71 + 41-); and MK-MEP (CD71 + 41+).

### Monocle trajectory analysis and sequential cultures identify a novel megakaryocyte-committed progenitor population

**Fig. 7:**
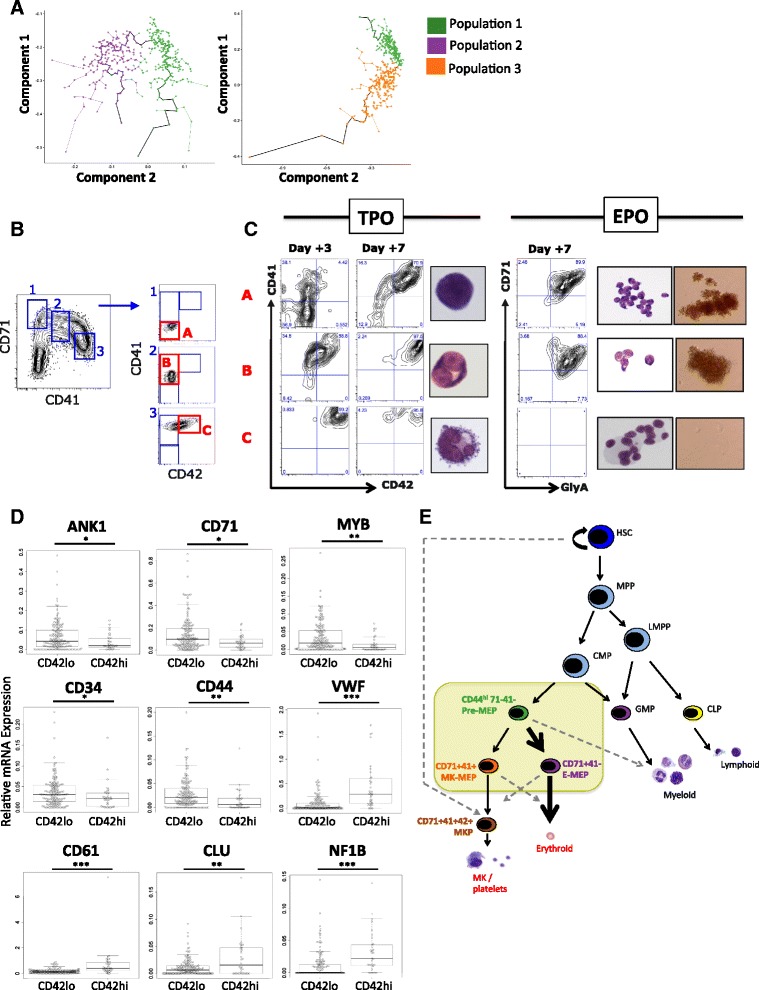
Monocle trajectory analysis and sequential cultures identify a novel megakaryocyte-committed progenitor population. **a** Pseudo-temporal ordering of cells using Monocle [37]. Trajectories are shown for Population 1 to 2 (Pre-MEP to E-MEP; *left plot*) and Population 1 to 3 (Pre-MEP to MK-MEP; *right plot*). **b** CD71 + 41- 42- (Population A), CD71 + 41 + 42- (Population B), and CD71^mid^CD41^hi^CD42+ (Population C) were FACS-isolated from day 4 in vitro megakaryocyte cultures for secondary culture and re-plated in either TPO-based (no EPO, *left*) or EPO-based (no TPO, *right*) cultures. **c** *Left*: 3 and 7 days after re-plating in TPO medium, Populations A, B, and C demonstrated progressive megakaryocytic maturity. Population A gave rise to CD41 + CD42-/+ megakaryocytes; **b** and **c** showed progressive CD42 acquisition supporting a unidirectional differentiation hierarchy. *Photographs* show representative cell cytospins 3 days after secondary culture. Population A shows early megakaryoblasts; Population B shows CD41+ CD42+/- megakaryocytes with single/bi-lobulated nuclei; Population C shows mature, proplatelet-forming megakaryocytes, multilobulated nuclei. *Right*: In parallel, Populations A, B, and C were re-plated into EPO-based secondary cultures (without TPO) and methylcellulose to test erythropoietic potential. A and B gave rise to CD71 + GlyA+ progeny with typical erythroblast morphology and BFU-Es; C were unable to differentiate into erythroid cells and were immunophenotypically/morphologically identifiable as CD41 + 42+ polyploid megakaryocytes (with abnormal nuclear lobe separation). n = 4. **d** Expression of selected genes in the MK-MEP subpopulation stratified according to CD42 cell surface expression. CD71 + 41 + CD42+ cells showed significantly lower expression of erythroid genes (e.g. *ANK1*, *CD71*, *MYB*), genes associated with more primitive HSC/progenitors (e.g. *CD34*, *CD44*) and higher expression of megakaryocyte genes (e.g. *VWF*, *CD61*, *CLU*, *NF1B*) than CD71 + 41 + CD42- cells. **e** A revised model of the megakaryocyte-erythroid differentiation hierarchy showing replacement of classically defined MEP with three novel subpopulations (*yellow box*). *Arrows* represent weighted differentiation capability; *dashed arrows* represent the potential for alternate-lineage differentiation. CLP, common lymphoid progenitors; CMP, common myeloid progenitors; E, erythroid; GMP, granulocyte/macrophage progenitors; HSC, hematopoietic stem cells; LMPP, lymphoid-primed multipotent progenitors; MEP, megakaryocyte-erythroid progenitors; MK, megakaryocyte; MPP, multipotent progenitors

Finally, we performed a monocle trajectory analysis [37] to obtain a pseudo-temporal ordering of single cells along their differentiation trajectory on the basis of their transcriptional profiles (Fig. 7a, Additional file 1: Figure S5A, B). Two separate trajectories were investigated, from Pre-MEP to E-MEP (Fig. 7a, left plot) and Pre-MEP to MK-MEP (Fig. 7a, right plot). Additional file 1: Figure S5A shows heatmaps illustrating how expression of selected genes changed with the pseudotime trajectories. Additional file 1: Figure S5B shows selected genes along the Pre-MEP to E-MEP and MK-MEP trajectories. This analysis illustrates downregulation of *CD44* and *CD34* together with upregulation of *GATA1* and *CD71* along both trajectories, in keeping with the more primitive phenotype of the Pre-MEP population, which retains myeloid potential. In contrast, a number of genes showed distinct erythroid or megakaryocyte-specific expression with progressive separation along each trajectory. For example, upregulation of *CNRIP1*, *KLF1*, and *LEF1* occurred along the E-MEP trajectory and *CD41*, *CD61*, *CD42*, *NF1B*, and *VWF* along the MK-MEP trajectory (Additional file 1: Figure S5A, B). Notably, *CD42* and *VWF* expression increased markedly along the MK-MEP trajectory and correlated with loss of erythroid gene expression such as *KLF1* and *CNRIP1* (Additional file 1: Figure S5B). As the CD42-positive cells also clustered at the apex of Population 3 in the PCA (Additional file 1: Figure S2A), we reasoned that CD42 surface expression might represent a marker of full commitment to the megakaryocyte lineage with associated loss of erythroid potential. To explore whether the expression of CD42, restricted to ~20 % of MK-MEP cells and <1 % of total MEP overall (Fig. 2g) was associated with definitive commitment to the megakaryocyte lineage, in vitro megakaryocyte liquid cultures were established from healthy donor Lin-CD34+ cells and defined megakaryocyte progenitor populations were isolated from day 4 cultures for secondary subcultures according to their surface CD71, CD41, and CD42 expression (Fig. 7b, Populations A, B, and C). In secondary cultures in TPO-based liquid culture, cell fractions A (CD71 + CD41- CD42-), B (CD71 + CD41 + CD42-), and C (CD71 + CD41 + CD42+) showed progressive megakaryocyte maturity by morphology and CD41CD42 co-expression after 3 and 7 further days of TPO stimulation (Fig. 7c). If switched to EPO-based medium and methylcellulose (without TPO) for secondary culture, Populations A and B gave rise to mature CD71^hi ^GlyA+ erythroblasts and erythroid colonies, while Population C had no erythroid potential (Fig. 7c, right panel). This confirmed that both CD71 + 41- 42- and CD71 + 41 + 42- populations (Populations A and B, Fig. 7b) contained cells with both megakaryocytic and erythroid potential, while CD71^mid^41 + 42+ co-expression marked the first identifiable lineage-committed MKP with complete loss of erythroid potential (Population C, Fig. 7b). In keeping with this, CD71 + 41 + CD42+ cells, compared to CD71 + 41 + CD42- cells, demonstrated significantly higher expression of megakaryocyte genes (e.g. *CD41*, *CD61*, *VWF*, *CLU*, *NF1B*) and significantly lower expression of erythroid-associated genes (e.g. *ANK1*, *CD71*, *MYB*). MYB is a transcription factor that enhances erythroid differentiation at the expense of megakaryopoiesis [38], in keeping with commitment to the megakaryocyte lineage.

## Conclusions

To date, the primary intermediate pathway in physiological human megakaryocyte and erythroid differentiation, the MEP, has yet to be well defined. In this study we applied single-cell approaches to investigate cellular heterogeneity among classically defined, immunophenotypic MEP in order to refine the cellular pathways leading to megakaryocyte and erythroid lineage commitment.

Single-cell transcriptional profiling of individual MEP cells with a known immunophenotypic profile demonstrated that the CD123-CD45RA- fraction of normal Lin-CD34 + CD38+ cells, previously classified as MEP, does not constitute a homogeneous population of progenitors. Rather, it is composed of at least three subfractions with distinct gene expression and functional capacities—cells enriched for erythroid/megakaryocytic output but with residual myeloid differentiation capacity (“Pre-MEP”), and erythroid-primed and megakaryocyte-primed, bipotent fractions (“E-MEP” and “MK-MEP”) (model, Fig. 7e). Further, considering the relative proportion of these cellular fractions (Fig. 2f) and their differentiation and proliferation efficiencies, we conclude that: (1) only a minority of classically defined MEP cells (~20 %) are truly bipotent cells with the capacity for megakaryocyte and erythroid differentiation as identified by in vitro assays; rather, the vast majority of single MEP cells preferentially generate colonies of exclusively erythroid or megakaryocytic progeny; and (2) there is an overall bias towards erythroid-only output from single MEP cells, as would be predicted by the observed frequencies of lineage-committed erythroid and megakaryocytic cells in normal bone marrow.

This work also demonstrates the power of integrating data from single-cell gene expression profiling and single-cell functional assays together with index sorting data to accurately map the cell surface immunophenotype of each individual sorted cell. As illustrated here, analysis of single-cell gene expression data using higher-level ordination techniques such as PCA may fail to identify less-frequent cellular subpopulations, that are nevertheless clearly distinct when selectively purified and analyzed in larger numbers.

Our findings are in keeping with recent insights from single-cell profiling of hematopoietic progenitor cells indicating that a large fraction of these cells are strongly biased towards specific lineage differentiation pathways rather than being multi/oligo-potent cells as suggested in the traditional hematopoietic hierarchical model [3, 7, 39, 40]. In addition, previous studies of murine bone marrow have demonstrated the existence of megakaryocyte-biased oligopotent megakaryocyte/erythroid progenitors [41] and multipotent progenitors [12, 42], as well as CD71 + CD105- erythroid-primed MEP in human samples [18]. However, this is the first report identifying human, megakaryocyte-biased, bipotent cells and suggesting a strategy for prospective isolation of all three MEP subpopulations and early lineage-committed MKP. Our studies were performed on samples from healthy apheresis donors who had received treatment to mobilize HSC/progenitors from the bone marrow to peripheral blood. As mobilization therapy does not alter the relative proportion of immunophenotypic MEP within the CD34+ cell compartment [43], we do not anticipate that this will impact on how generalizable our findings will be to other physiological hematopoietic progenitor populations, but this will nevertheless be an important factor consider when applying this novel cell-sorting approach in future studies of MEP. While our study provides insights into the heterogeneity of human MEP using a panel of selected genes, advances in whole transcriptome analysis now allow unbiased analysis of the transcriptome of single cells. Such studies might provide additional insights into novel regulatory pathways and deterministic factors that might govern erythroid versus megakaryocyte commitment.

These observations redefine the cellular hierarchy underlying erythroid and megakaryocyte lineage commitment, and will enable a more precise molecular investigation of normal and perturbed erythro-megakaryocyte differentiation, such as in myeloproliferative neoplasms where a pathognomonic increase in megakaryocyte progenitors occurs, often coupled with marked anemia, and in erythro-myeloid leukemias. This work may also assist in strategies aimed at developing expanded erythroid and megakaryocyte/platelet populations as cellular therapy.

## Methods

### Sample preparation

CD34+ cells were collected by apheresis from 14 healthy donors treated with G-CSF to mobilize hematopoietic stem/progenitor cells from bone marrow to peripheral blood. These samples were obtained from the National Institutes of Health Cell Processing Laboratory and the Stem Cell Laboratory, Hammersmith Hospital, Imperial College NHS Trust UK. Cryopreserved aliquots of CD34+ cells were thawed, washed in RPMI + 10 % FBS, and transferred to PBS + 2 mM EDTA + 1 % FBS. The mean age of the donors was 45 years (range, 23–68 years); 11/14 were male. There were no significant differences in the proportion of MEP as a % of Lin- CD34 + CD38+ cells nor of the MEP subpopulations between male and female donors, or between donors younger and older than 45 years of age.

### Flow cytometric analysis and sorting

A panel of nine fluorophore-conjugated monoclonal antibodies plus a live/dead stain was used for flow cytometric cell sorting and analysis as follows: CD34-PE Cy7 clone 4H11 (eBiosciences); CD38 Alexa Fluor 700 clone HIT2 (eBiosciences); Lineage-APC (containing antibodies against: CD2, CD3, CD14, CD16, CD19, CD56, and CD235a, eBiosciences); CD123-BV605 clone 73G (BD Biosciences); CD45RA-APC eFluor 780 clone HI100 (eBiosciences); CD71-FITC clone OKT9 (eBiosciences); CD41-efluor 450 clone HIP8 (eBiosciences); CD42-PE clone HIP1 (eBiosciences) and CD36 PerCP-Cy5.5 clone CB38 (BD Biosciences). When CD44 was included in the immunophenotyping panel, CD36 PerCP-Cy5.5 was replaced with CD44 PerCP-Cy5.5 clone IM7 (eBiosciences). Cells were stained with specific antibodies for 20 min at 4 °C, and washed prior to staining with Live/Dead Fixable Aqua Dead Stain Kit (Life Technologies) for 30 min at room temperature. Cells were washed and analyzed on a FACSAria III (BD Biosciences) using FACSDIVA™ software v. 8.0.1. Gates were set using strict fluorescence-minus-one controls run for each sample and experiment. Cell doublets, non-viable (AQUA positive), and lineage-positive cells were excluded. Data were analyzed on FlowJo software v. 9.8.2 (Tree Star).

### Multiplex, single-cell high throughput microfluidic real-time PCR

A total of 807 single cells were directly isolated from three healthy apheresis donors (199 MEP cells plus 70 CD71 + 41+ MEP from each donor). Cells were isolated by index-FACS into each well of 96-well PCR plates containing 5 μL lysis buffer (CellsDirect One-Step qRT-PCR kit [Invitrogen], SUPERASE-In RNase inhibitor [Ambion], TE buffer) and 0.2× Taqman assay mastermix. Plates were sealed, vortexed, briefly centrifuged and then cDNA synthesis and sequence-specific preamplification performed (reverse transcriptase – 50 °C, 15 min; inactivation at 95 °C, 2 min; specific target amplification 95 °C 15 s then 60 °C 4 min, 22 cycles). Plates were stored at –20 °C until analysis. Preamplified products were diluted fourfold prior to analysis. Samples were analyzed using Universal PCR Master Mix (Applied Biosystems) and individual Taqman gene expression assays (Life Technologies, Additional file 2: Table S1), on the Biomark System (Fluidigm) using the 96.96 Dynamic Arrays as per manufacturers protocol. Data were analyzed using Fluidigm RT-PCR Analysis Software to standardize thresholds for each gene assay across plates.

The assays were validated by titration to confirm linearity. Eight fourfold serial dilutions were made from three pools of bulk-sorted cells with technical triplicates at each dilution. Missing values and those with Ct >40 were removed and technical replicates were subsequently merged. Normalization of individual pools was performed by using a linear model with the formula Ct.value ~ 0 + dilution + pool to estimate and remove the effect of the different starting concentrations. Log_2(dilution)_ was plotted against Ct value and linear models were fitted for each assay to obtain a coefficient of dilution variable and r^2^ measure of goodness of fit. The model formula was Ct.value ~ dilution. These analyses showed that all assays in the panel demonstrated good linearity (Additional file 2: Table S1).

To validate that the addition of CD44 to the immunophenotyping panel identified the same MEP subpopulations as seen on PCA of the single-cell data, 100 cells from the three MEP subpopulations (CD44^hi^ CD71- 41-, CD71 + CD41-, and CD71 + CD41+) were sorted according to the strategy in Fig. 4c into wells of a 96-well PCR plate in triplicate from each of four donors. Cell lysis, preamplification, and gene expression profiling was then performed as described above for the single cells (with a reduction in preamplification cycles from 22 to 16). Missing values and Ct > 40 were removed and technical replicates merged. Normalization was performed to the mean of B2M and GAPDH (as per the single-cell analysis). Boxplots were prepared in R. *P* values were generated using two-sample two-sided T-test.

### qRT-PCR data analysis

Data analysis was performed in R (version 3.2.2). Ct values of all assays marked as “Fail” by the instrument were set to the limit of detection value (Ct = 40). Ct values beyond the limit of detection were set to that value. Non-informative assays with standard deviation of zero were excluded (n = 3). Some gene assays were included in duplicate for quality control; data from these genes were merged or removed, resulting in data for 87 gene expression assays being included in the analyses (Additional file 1: Figure S1B). For duplicated assays where one duplicate failed, data were not merged and only one replicate kept. For duplicated assays where good correlation was observed between duplicates, the data were merged. Gene assays not included in duplicate were removed if zero variance or the expected distribution of Ct values was not seen.

Cells were excluded if more than 70 assays did not result in amplification in that cell. Cells that displayed low levels of B2M or GAPDH were excluded, using cutoffs of 13 and 15 Ct cycles, respectively. The cutoff values were selected after inspection of individual histogram plots to identify outliers. Finally, the mean Ct for each cell was calculated including Ct values for all assays that yielded detectable amplification, and cells that displayed mean Ct value greater than 20 were removed after visual inspection of the data to exclude outliers. After applying these stringent technical exclusion criteria, 681 cells were included in analyses (489 unfractionated MEP cells and 192 71 + 41+ MEP cells). Ct values were normalized to the mean of B2M and GAPDH expression. Normalized Ct values and raw data are listed in Additional file 2: Table S2 and S3, respectively.

PCAs were performed in R using the prcomp function. Spearman correlation coefficients were calculated in R. Monocle trajectory analysis was performed using the monocle R package (version 1.2.0) [37]. 2^-(Normalized Ct)^ expression values were used for the analysis and housekeeper genes were excluded. Network visualization of gene correlation was performed in Cytoscape (version 3.2.1) [44]. t-SNE analysis [27–30] was performed on the normalized Ct Values in MATLAB (version R2015a).

### Data availability

The raw data have been deposited in Gene Expression Omnibus under accession number GSE79331 and are provided in Additional file 2: Table S3.

### Robustness of PCA clustering by gene permutation

In order to assess the sensitivity of the PCA to gene permutation, the stability of cell-to-cluster assignment was assessed for subsets of the assayed genes of cardinality 10 to 87. For each gene set size, up to 10,000 distinct subsets of genes (or fewer where 10,000 distinct permutations were not possible) were selected and PCA was repeated. Cells were assigned to one of three clusters using k-means clustering on the basis of the first two PC values. Correspondence of the clusters defined by k-means clustering to the clusters defined in Fig. 3b was determined by obtaining the mode of the original cluster assignments in each new cluster. The percent of cells assigned to the same cluster as in the original analysis was then assessed. Furthermore, for each permutation a random assignment of cells to clusters was performed and congruency of the assignments to this cell population was also assessed in order to evaluate the correct assignment due to chance.

### ZIFA analysis

Visualization of the expression data was performed using the ZIFA tool [31] which accounts for and models the drop-outs in single-cell analyses. ZIFA requires that the input drop-out values are represented by values close to 0. Normalized Ct values were transformed so as to satisfy this requirement. The maximal non-drop-out Ct value per gene was identified (Ct_max_gene_) and all drop-out values were set to two cycles beyond this gene-specific limit of detection. A transformation inverting the [0,Ct_max_gene_] interval for each gene indvidually was then applied to the Ct values before the data were used as input for ZIFA. This ensured that all drop-outs were correctly treated by ZIFA.

### Clonogenic assays in semi-solid medium

Clonogenic assays were performed on FACS-isolated cells using Methocult H4034 to support erythroid and myeloid colonies and Megacult to examine megakaryocyte/myeloid colony formation (both Stemcell Technologies). To assess erythroid/myeloid colony forming capacity, single cells were directly sorted into individual wells of flat-bottomed 96-well plates containing 100 μL Methocult, briefly centrifuged, and incubated at 37 °C in a fully humidified 5 % CO_2_ atmosphere. Colonies were scored and photographed using an inverted microscope (Zeiss Axio Observer) at days 7 and 10–14, as per established criteria [45]. Erythroid colonies were easily identified by their red color and “burst” morphology (Fig. 6a) and megakaryocyte and myeloid colonies by their characteristic size and morphology [46]. Some individual colonies were plucked from the semi-solid medium under direct light microscopy on days 12–14, washed, and analyzed by flow cytometry for expression of lineage-associated surface antigens. Erythroid cells were identified as CD71 + GlyA+; myeloid as CD11b/CD14+; and megakaryocytic as CD41 + CD42+. Single-cell Megacult assays are not possible because of the lower clonogenicity associated with megakaryocyte colony formation. Therefore, to determine megakaryocyte colony-forming capacity, MEP subfractions were FACS-isolated as bulk preparations into 1.5-mL tubes containing 50 μL Iscove’s Modified Dulbecco’s Medium with 1 % Pen-Strep and 40 μg/mL human LDL (Stemcell Technologies) and plated in Megacult medium + collagen as per manufacturer’s protocol in double chamber slides, and incubated at 37 °C, 5 % CO_2_ for 8–10 days. Megakaryocyte colonies were enumerated after fixing and staining with anti-human GPIIb/IIIa (Stemcell Technologies Megacult staining kit for CFU-MK) and counterstained with Evans Blue. Using this method, megakaryocyte and myeloid (GEMM/GM) colonies are easily distinguished by their pink and blue stains, respectively. Colony number per 1000 cells sorted/chamber was calculated.

### Single-cell liquid culture assay

Single cells were seeded by index-FACS into individual wells of round-bottomed 96 well plates containing 70 μL of serum-free media containing the appropriate cytokines to support erythroid (EPO, SCF, IL3, IL6) [17, 25, 47] and megakaryocyte (TPO, SCF, hLDL) [48] differentiation. Stemspan (Stemcell Technologies) was supplemented with these cytokines at the following conentrations: TPO (100 ng/mL); SCF (100 ng/mL); IL3 (10 ng/mL); IL6 (10 ng/mL); hLDL (40 mg/mL); 1 U/mL EPO. All cytokines were from Peprotech; hLDL was from Stemcell Technologies. Wells were inspected by light microscopy 6 days after seeding and photographed using a Zeiss Axio Observer microscope to determine cell number and morphology to identify the presence of characteristic erythroblasts and proplatelet-forming megakaryocytes (Fig. 6c). At this time point, MEP-derived MKP undergo pro-platelet formation, but minimal apoptosis, and have acquired high levels of surface CD41 and CD42 allowing their distinction immunophenotypically from EP which are CD71^hi^ CD42 negative (Additional file 1: Figure S4D).

For lineage determination by flow cytometry, cells were immunostained directly in the well to assess surface expression of lineage markers. To analyze wells by flow cytometry, 40 μL media was carefully removed from the top of each well, and 20 μL FACS buffer containing a cocktail of antibodies was added to each well (CD71-FITC; CD41-PeCy7; CD36 PerCPCy5.5; CD42-PE; GlyA-Pacific Blue; CD11b/CD14-APC). Plates were incubated for 20 min at 4 °C, cells washed and analyzed on a LSR cytometer either manually in microeppendorf tubes placed inside 5 mL FACS tubes or the entire plate analyzed using an automated high-throughput sampler. Erythroid cells were identified as CD71^hi^ CD36^hi^ CD41- CD42- ; megakaryocytes identified as CD71^mid^ CD36^mid^ CD41+ 42+; and myeloid cells as CD11b/CD14+ (no myeloid cells observed).

To analyze wells by fluorescence microscopy, single cells were seeded into individual wells of 384-well Optical Bottom Plates (Nunc) containing 70 μL bipotent differentiation medium as described above. After 6 days of incubation, 40 μL media was carefully removed and 20 μL buffer containing CD71-FITC and CD41-APC was added. Plates were incubated for 20 min at 4 °C, centrifuged briefly, and then imaged on a Zeiss Spinning Disk Confocal microscope.

### Determining the differentiation potential of in vitro cultured megakaryocyte progenitor subsets in sequential assays

CD34+ cells from mobilized, healthy donors were plated in Stemspan (Stemcell Technologies) at 1–5×10^4^ cells/mL with 1 % pen-strep and 10 μL/mL megakaryocyte expansion cocktail containing TPO, SCF, IL6, IL9 (Stemcell technologies), and hLDL. On day 4, MKP subfractions were FACS-isolated according to their expression of CD71 and CD41. At this stage, 85–95 % of MKP remain CD34+. A total of 3000 cells from CD71 + 41- 42-, CD71 + 41 + 42-, and CD71 + 41 + 42+ subsets were sorted into 1.5-μL tubes containing Stemspan, centrifuged, and the pellet resuspended in either erythroid (EPO, SCF, IL3, IL6) or megakaryocyte-specific (TPO, SCF, IL6, IL9, hLDL) medium; single-cell clonogenic assays in Methocult H4034 were performed as described above. An aliquot of cells from each well was removed for FACS analysis 3, 6, and 10 days after FACS isolation and cultures replenished with fresh medium.

Cultured cells were cytocentrifuged at 4000 rpm for 5 min onto Superfrost slides, using a Shandon Cytospin 2 (Fisher Scientific) followed by methanol fixation and staining with May-Grunwald Giemsa.

### Other statistical analyses

Data analysis was performed using Excel and Prism 6 software; graphs drawn using Prism. Mean ± SEM are presented. Unpaired T-tests or ANOVA were performed for the FACS data analysis and differentiation assays as appropriate and as indicated in figure legends. For qRT-PCR data and index sorting data, *P* values were calculated using the non-parametric Kolmogorov-Smirnov (KS) test and adjusted to q-values using false discovery rate (FDR) correction to account for multiple testing [49].

### Ethics approval

All donors provided informed consent and samples were collected and utilized under approval by Imperial College Healthcare NHS Trust Tissue Bank (Imperial College, London, UK) and National Institutes of Health Research Ethics Office approval (NIH, Bethesda, USA) (94-I-0073). All experimental methods comply with the Helsinki Declaration.

## Additional files

Additional file 1: Supplementary Figures S1 - S5. (PDF 10849 kb) Additional file 2: Supplementary Tables S1 - S3. **Table S1.** Gene assays included in multiplex RT-PCR analysis with coefficient of dilution (R2) examined over fourfold serial dilutions with technical triplicates at each dilution. **Table S2.** Normalized Ct values for gene expression data for all genes and cells. **Table S3.** Raw data for single-cell gene expression analysis (non-normalized Ct values). (XLSX 1059 kb)

## Abbreviations

CFU: colony forming unit
CLP: common lymphoid progenitors
CMP: common myeloid progenitors
E: erythroid
EB: mature CD71 + GlyA+ erythroblasts
EP: CD71 + CD36+ early erythroid progenitors
FACS: fluorescence-activated cell sorting
FDR: false discovery rate
GMP: granulocyte/macrophage progenitors
HSC: hematopoietic stem cells
KS: Kolmogorov-Smirnov test
Lin: lineage
LMPP: lymphoid-primed multipotent progenitors
MEP: megakaryocyte-erythroid progenitors
MK: megakaryocyte
MPP: multipotent progenitors
PC: principal component
PCA: principal component analysis
qPCR: quantitative polymerase chain reaction
tSNE: t-distributed stochastic neighbor embedding
ZIFA: Zero inflated factor analysis
