# Supplementary material for: Single-cell profiling of human megakaryocyte-erythroid progenitors identifies distinct megakaryocyte and erythroid differentiation pathways
Source: Genome Biol. 2016 May 3;17:83. doi: 10.1186/s13059-016-0939-7 (PMC4855892; doi:10.1186/s13059-016-0939-7)
Supplement: Additional file 1: — Supplementary Figures S1 - S5. (PDF 10849 kb) [file 13059_2016_939_MOESM1_ESM.pdf]

SUPPLEMENTARY FIGURES

Supplementary Figure 1. Transcriptional and cell surface profile of the three MEP cellular subfractions.

S1A. Example sorting gates used to isolate common myeloid progenitors (CMP), granulocyte/macrophage progenitors (GMP) and megakaryocyte-erythroid progenitors (MEP). MEP gate was set below the fluorescence-minus-one (FMO)-defined negative CD123 population. **S1B.** % Variance explained by all principal components (PC). **S1C-F.** Distribution of the populations 1-3 and population 0 (unassigned cells) shown by sort plate (p1, p2, p3) (S1C, S1E) and apheresis donor (a1, a2, a3) (S1D, S1F) demonstrating no significant plate or sample effect. No important difference in Population 1/Population 2 ratios per plate was observed, given the overall ratio within each sample (P-values ranging 0.20 – 1.0 for all 9 plates).

Supplementary Figure 2. Localization of CD71+41+ and CD42+ MEP cells on the PCA, and confirmation of the PCA-defined MEP subpopulations by t-SNE, clustering by permutation and ZIFA analyses

S2A. CD42 surface expression (log_2 mean fluorescence intensity, MFI) overlaid on the PCA shown in Fig.2A. Red - high expression; Blue - low CD42 expression. CD42hi cells primarily locate to the lowest apex of Population 3. Circles represent CD71+CD41+ selected MEP cells and triangles represent unselected MEPs. **S2B.** t-SNE visualization of the single-cell expression data colored by the original population assignments from PCA shows highly similar clustering. Population 0 = unassigned cells. **S2C.** Sensitivity of the PCA to the selection of input genes was assessed by permutation analysis. PCA was performed using subsets of variable numbers of genes. The proportion of cells assigned to the original cluster was recorded for each permutation. Red data points indicate the mean proportion of cells assigned to the same cluster (y-axis) across all permutations of a particular size (x-axis). Blue data points evaluate congruent cluster assignment against random cell assignments. Each data point is based on a maximum of 10,000 different permutations (or fewer where the number of

possible permutations was limited). The cluster assignment of the cells is robust to the selection of genes – on average ~75% of cells are correctly assigned with as few as 25 genes. **S2D.** Two-dimensional visualization of expression data using ZIFA to model zero-inflation also distinguishes three subpopulations in agreement with PCA. **S2E.** Heatmap of gene expression in 681 single cells showing all 87 genes analyzed. Cells are grouped by the three populations as assigned by PCA (Fig. 2B). **S2F.** Hierarchical clustering of gene expression profiles also supported the division of Lin-CD34+CD38+CD123-CD45RA- MEP into three subpopulations as defined by PCA (Fig. 2B). Population 1 – green; Population 2 – purple; Population 3 – orange.

Supplementary Figure 3. Correlation between cellular immunophenotype as defined by index-sorting data with gene expression profiles.

S3A. Scatter plot representation of the confusion matrix describing performance of FACS classification of the cells to the PCA-assigned populations as shown in Fig.2B ([0] – unassigned; [1] CD71-41- (green box) [2] CD71+41- (purple box) [3] 71+41+ (orange box). Sensitivity and specificity for FACS immunophenotype in allocating cells to PCA population is tabulated below. **S3B.** Relative gene expression assayed by single-cell multiplex qRT-PCR correlated with surface antigen mean fluorescence intensity (MFI) determined by index FACS-sorting. Table shows the significance of the correlation. Fewer than 10 outliers were removed for the plots but these cells were retained in the calculations. **S3C.** CD44 and CD71 co-expression on MEP, CMP and GMP (top row) and the three MEP subpopulations (1) CD71-41-, (2) CD71+41- and (3) CD71+41+ (bottom row) shows that the CD71 and CD44 co-expression pattern of CD71- MEP (Population 1) is similar to CMP and GMP. **S3D & S3E.** Validation that the sorting strategy using CD44 as shown in Fig. 4C identifies the three MEP subpopulations seen on PCA of the single-cells. Three technical replicates of 100 cells were analyzed for each population from 4 healthy donors. Data shows relative mRNA expression of the surface antigens **(S3D)** used to identify the populations Pre-MEP, E-MEP and MK-MEP and also transcriptional profiles for lineage-affiliated genes **(S3E)** confirming findings at the single-cell level (*-p <0.05; **-p <0.01; ***-p <0.001)

Supplementary Figure 4. Single-cell differentiation assays demonstrate biased lineage differentiation of the three MEP subfractions corresponding to their transcriptional priming

S4A. Colony output and phenotype as a percentage of the number of single cells seeded from each MEP subpopulation in methylcellulose. (CFU – colony-forming unit; MK – megakaryocyte; E – erythroid; GM – granulocyte-macrophage; GEMM – granulocyte erythroid megakaryocyte macrophage; BFU – blast forming unit). P values shown in figure legend are for 1-way ANOVA comparison between the three populations. **S4B.** Colony output of single-cells isolated from CMP, GMP fractions[20] and MEP subpopulations. Classically-defined MEP ("Total MEP") are enriched for erythro-megakaryocytic differentiation but contain cells with residual myeloid activity (~10% output). Further, CD44^{hi}71-41- Pre-MEP are enriched for erythro-megakaryocytic activity as compared to CMP but also account for almost all of the residual myeloid potential observed when sorting individual cells from the total Lin-CD34+CD38+CD123-CD45-RA MEP fraction. **S4C.** Mean fluorescence intensity (MFI) of surface CD44 **(top chart)** and surface CD123 **(lower chart)** for cells generating erythroid/megakaryocyte, myeloid (GM or GEMM) or no colonies in methylcellulose. Data shown for 250 colonies derived from single MEP cells in 4 separate experiments. **S4D.** Example plots illustrating flow cytometric analysis of wells 6-days after seeding with single cells grown in media to support both erythroid and megakaryocyte differentiation. Examples of mixed colonies (top row), megakaryocyte (MK) only (middle) and erythroid (E) only progeny are shown. Erythroid cells are identified as CD71+CD42- (top and bottom rows, left panel). Megakaryocytes are identified as CD41+42+ (top and middle rows, right panel).

Supplementary Figure 5. Monocle trajectory analysis suggest a novel megakaryocyte-committed progenitor population

S5A. Heatmaps showing dynamic expression of selected genes across cells ordered in pseudotime for Pre-MEP to E-MEP trajectory (top heatmap) and Pre-MEP to MK-MEP (lower heatmap). Populations are indicated by green (Pre-MEP), purple (E-MEP) and orange (MK-MEP) bars below the heatmaps. **S5B.** Pre-

MEP to E-MEP (left plots) and Pre-MEP to MK-MEP (right plots) trajectories for relative mRNA expression of individual genes *CD34, CD44, GATA1, FLI1, CD42, VWF, KLF1* and *CNRIP* against pseudotime are shown.

Suppl. Figure 1

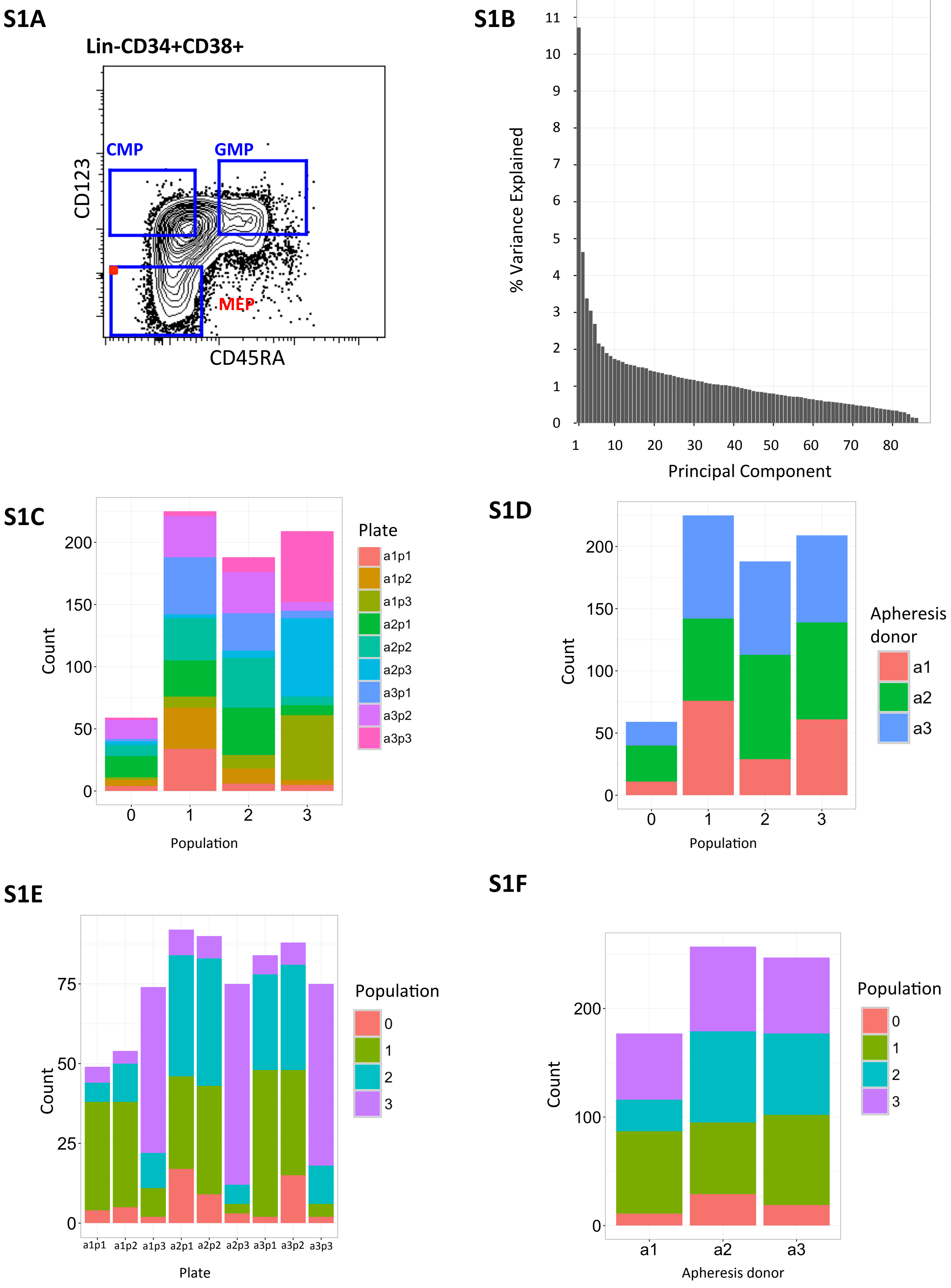

Suppl. Figure 2

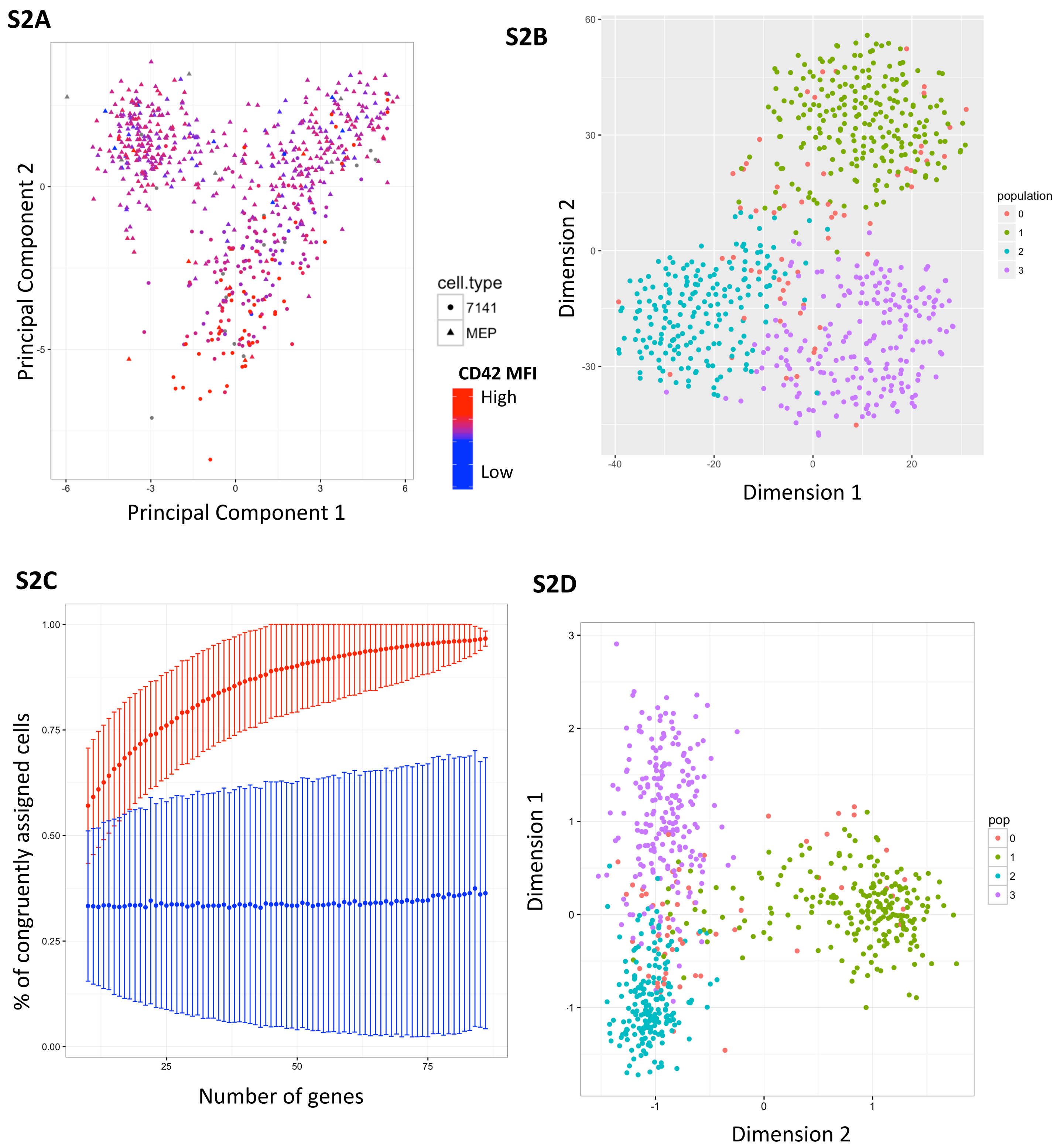

Suppl. Figure 2

S2E

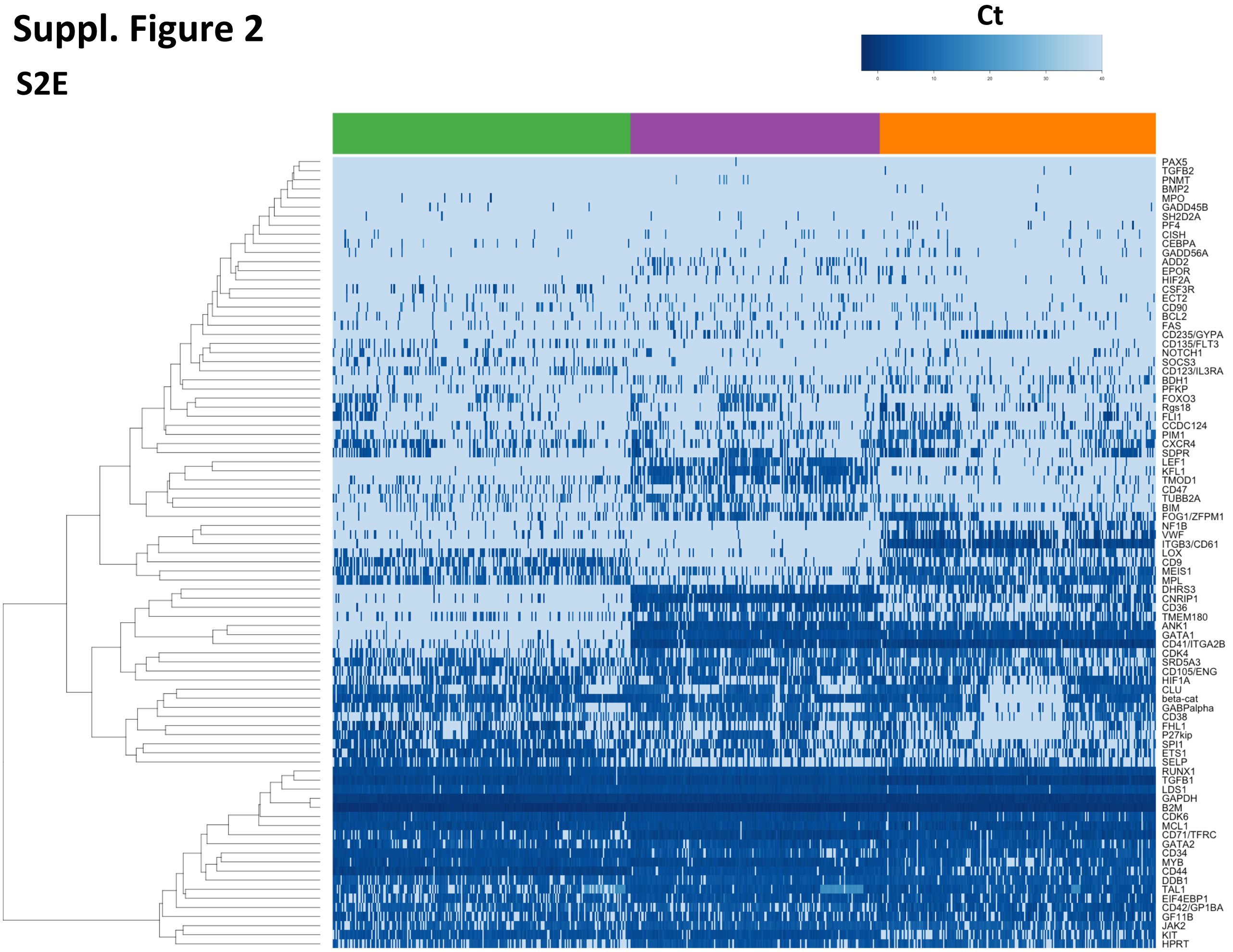

S2F

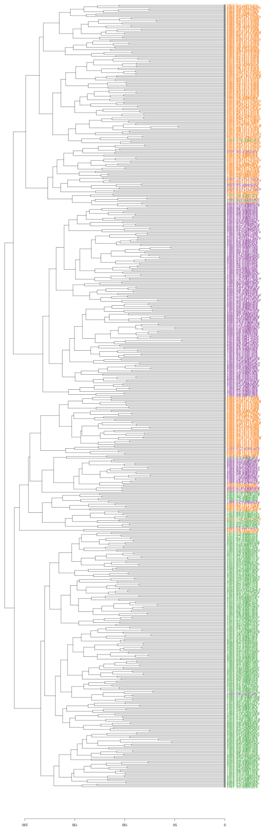

Suppl. Figure 3

S3A

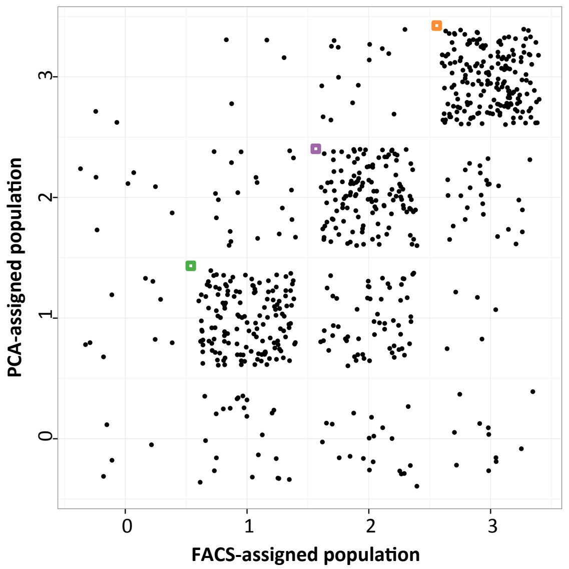

Population	1	2	3
Sensitivity	0.67	0.70	0.90
Specificity	0.89	0.81	0.91

Suppl. Figure 3

S3B

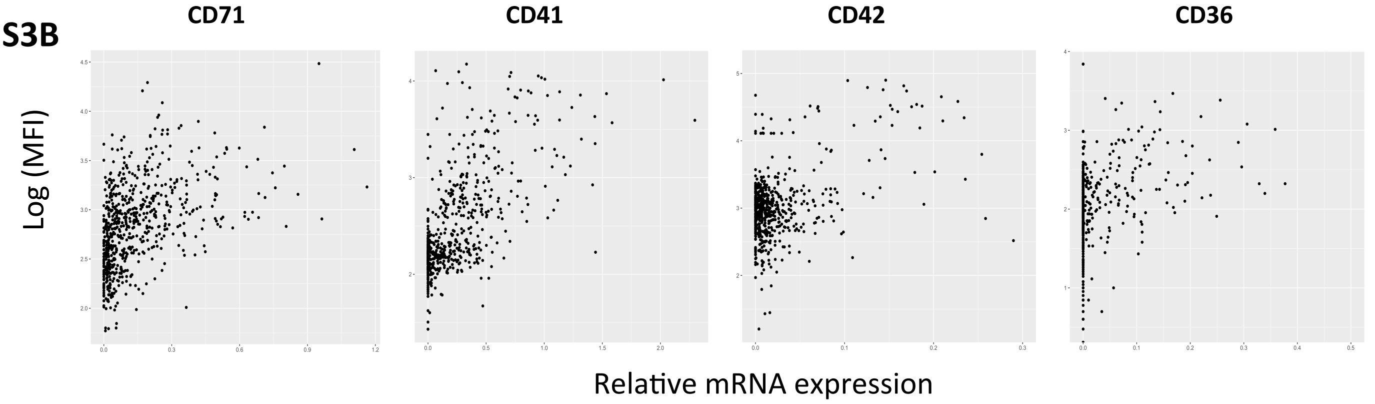

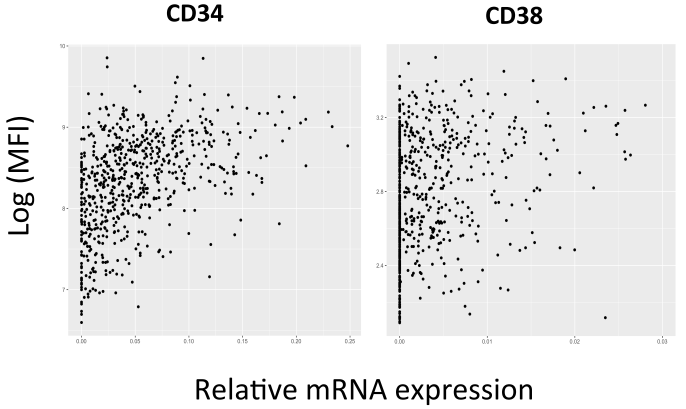

Surface antigen	Significance of Correlation
CD71	$<2.2 \times 10^{-16}$
CD36	1.44×10^{-9}
CD38	4.82×10^{-9}
CD41	$<2.2 \times 10^{-16}$
CD42	$<2.2 \times 10^{-16}$
CD34	$<2.2 \times 10^{-16}$

S3C

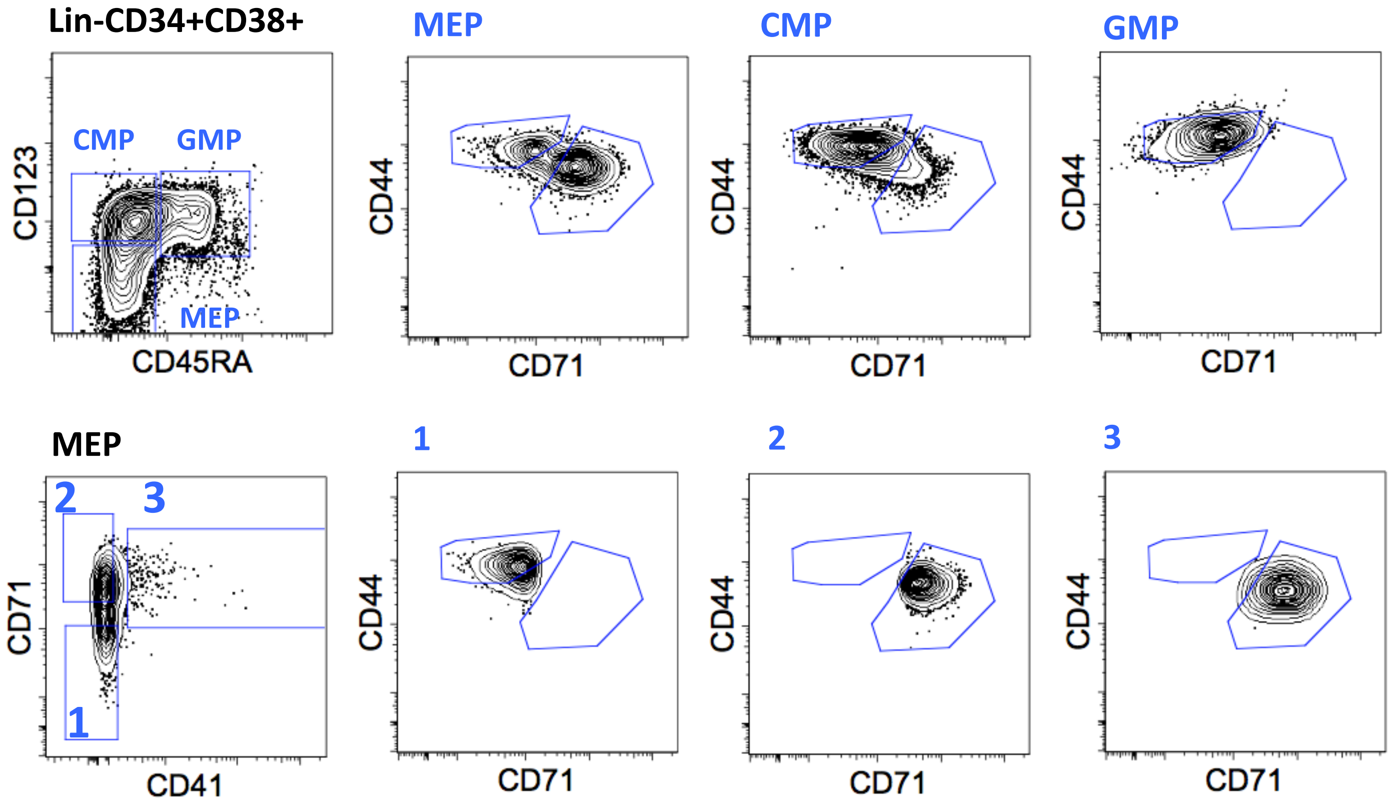

Suppl. Figure 3

S3D

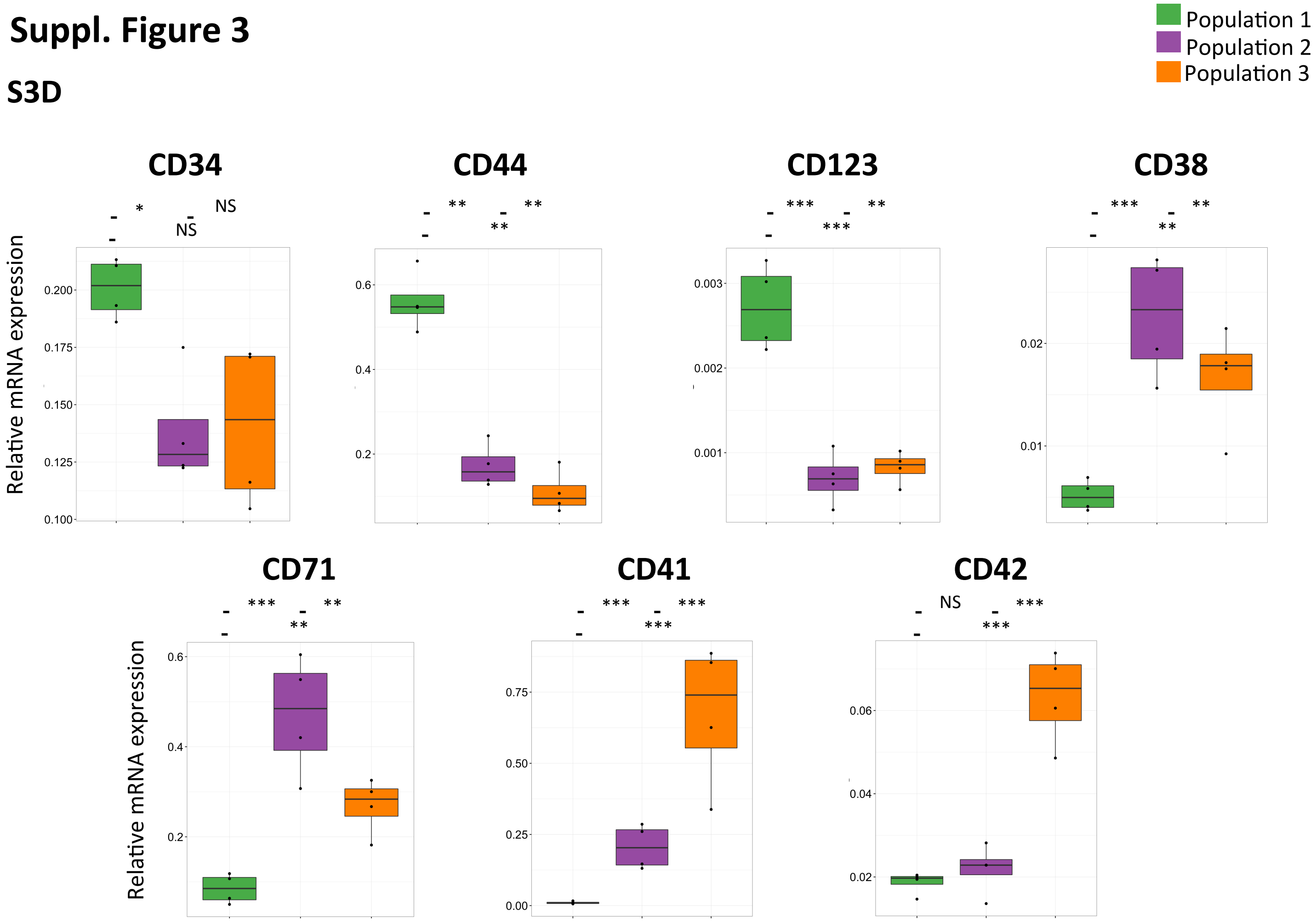

Suppl. Figure 3E

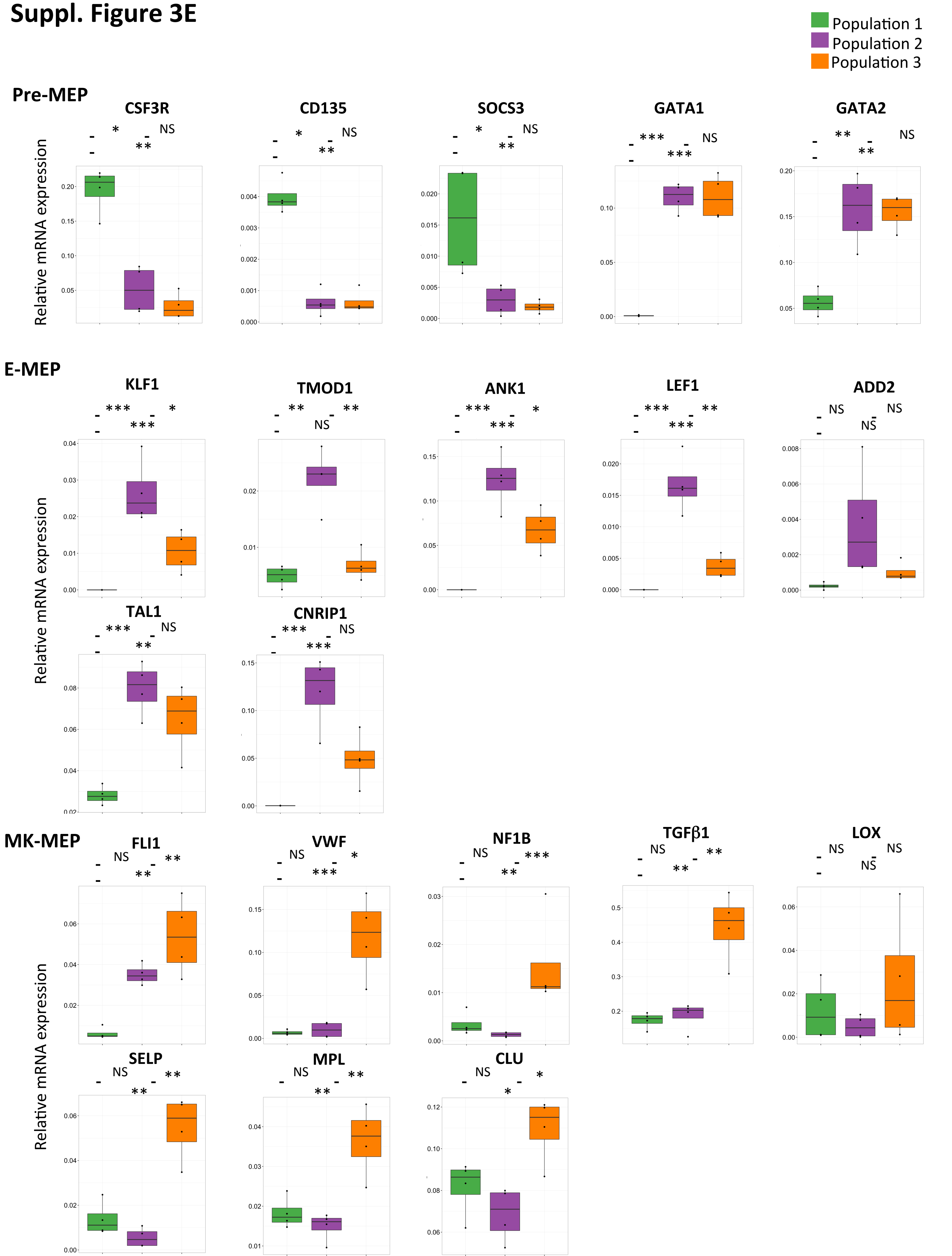

Suppl. Fig. 4

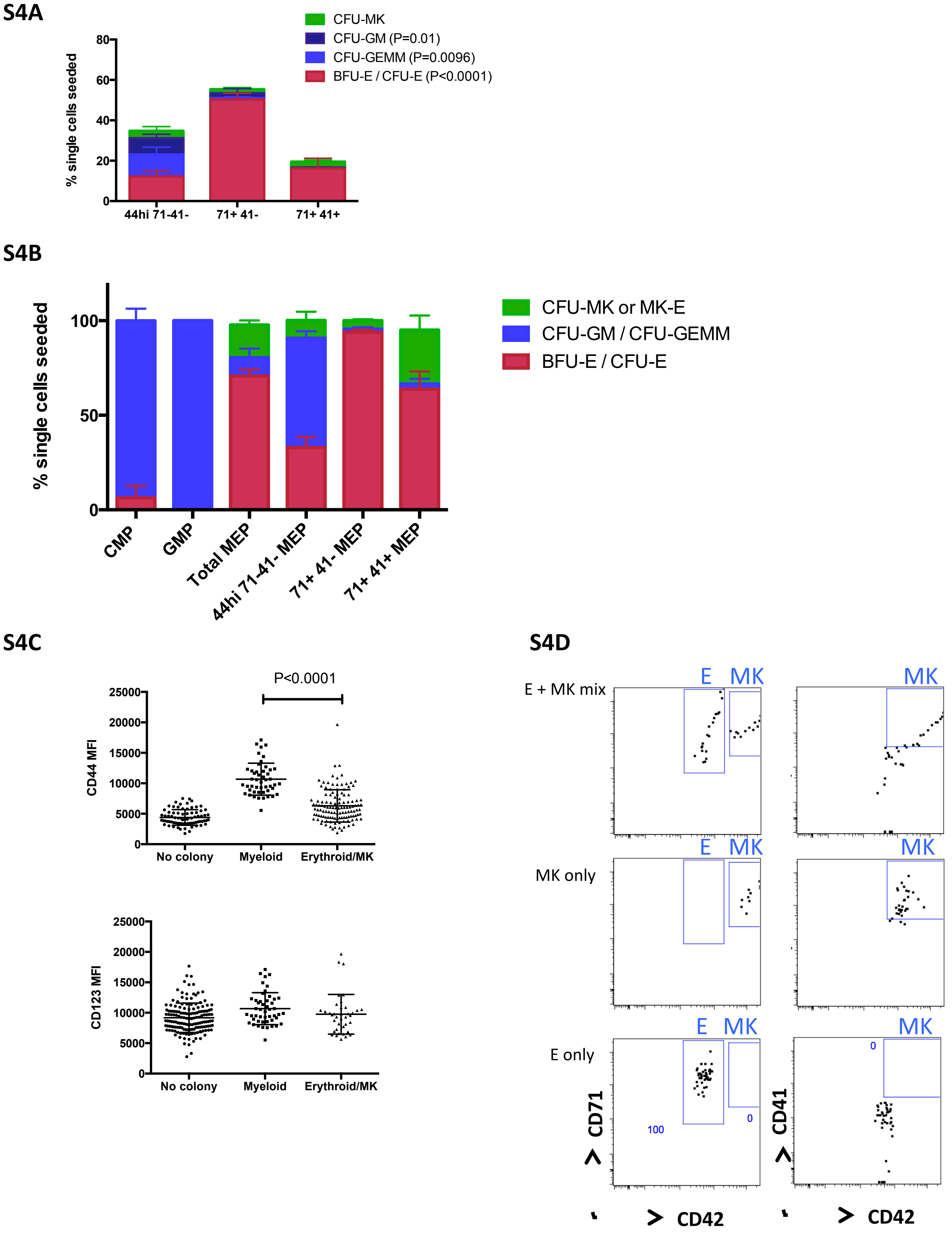
S4A
CFU-MK
CFU-GM (P=0.01)
CFU-GEMM (P=0.0096)
BFU-E / CFU-E (P<0.0001)
% single cells seeded
80
60
40
20
0
44hi 71-41-
71+ 41-
71+ 41+
S4B
CFU-MK or MK-E
CFU-GM / CFU-GEMM
BFU-E / CFU-E
% single cells seeded
100
50
0
CMP
GMP
Total MEP
44hi 71-41- MEP
71+ 41- MEP
71+ 41+ MEP
S4C
P<0.0001
CD44 MFI
25000
20000
15000
10000
5000
0
No colony
Myeloid
Erythroid/MK
CD123 MFI
25000
20000
15000
10000
5000
0
No colony
Myeloid
Erythroid/MK
S4D
E + MK mix
MK only
E only
E
MK
100
0
CD71
CD41
CD42

Suppl. Fig. 5

S5A

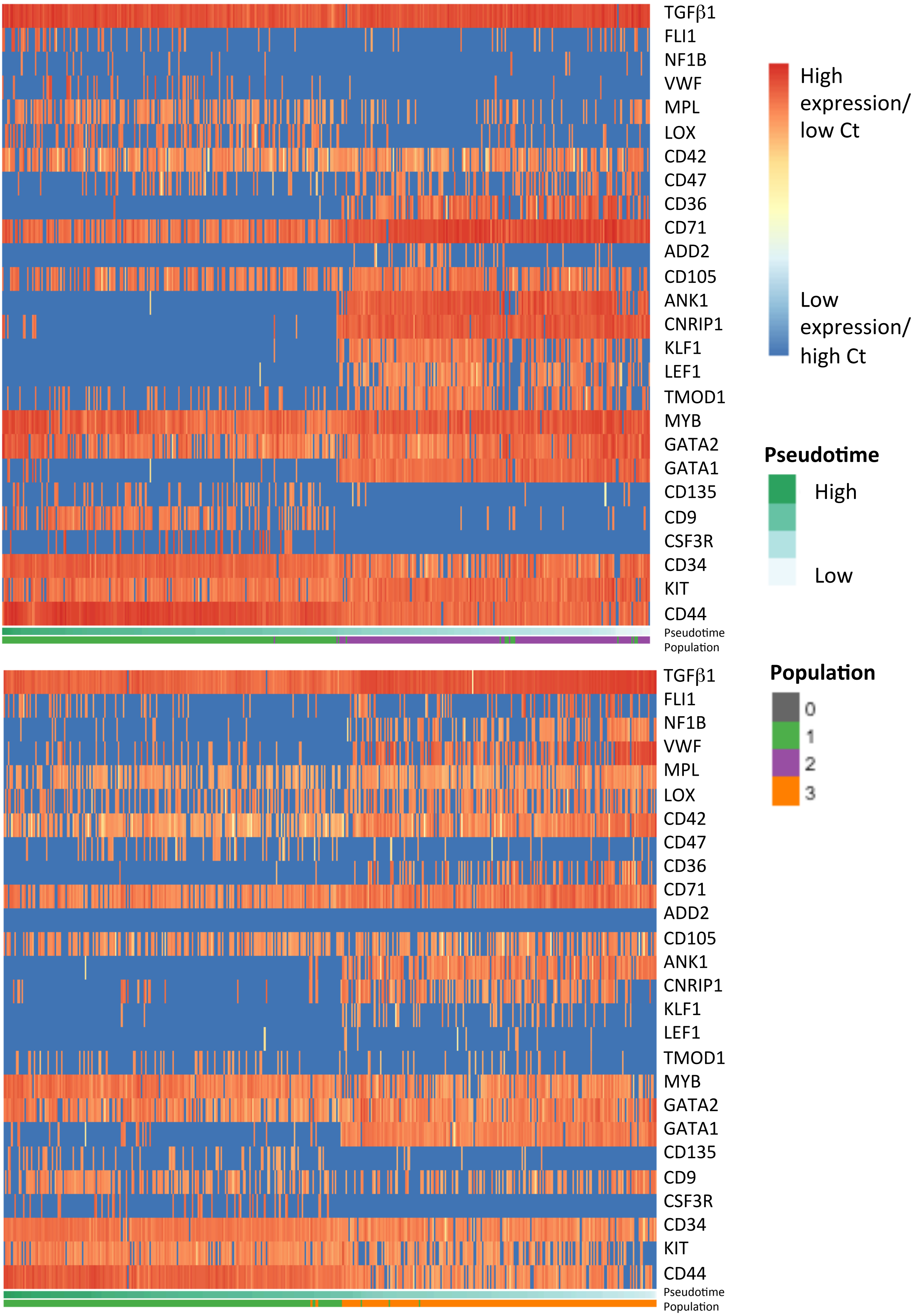

Suppl. Fig. 5

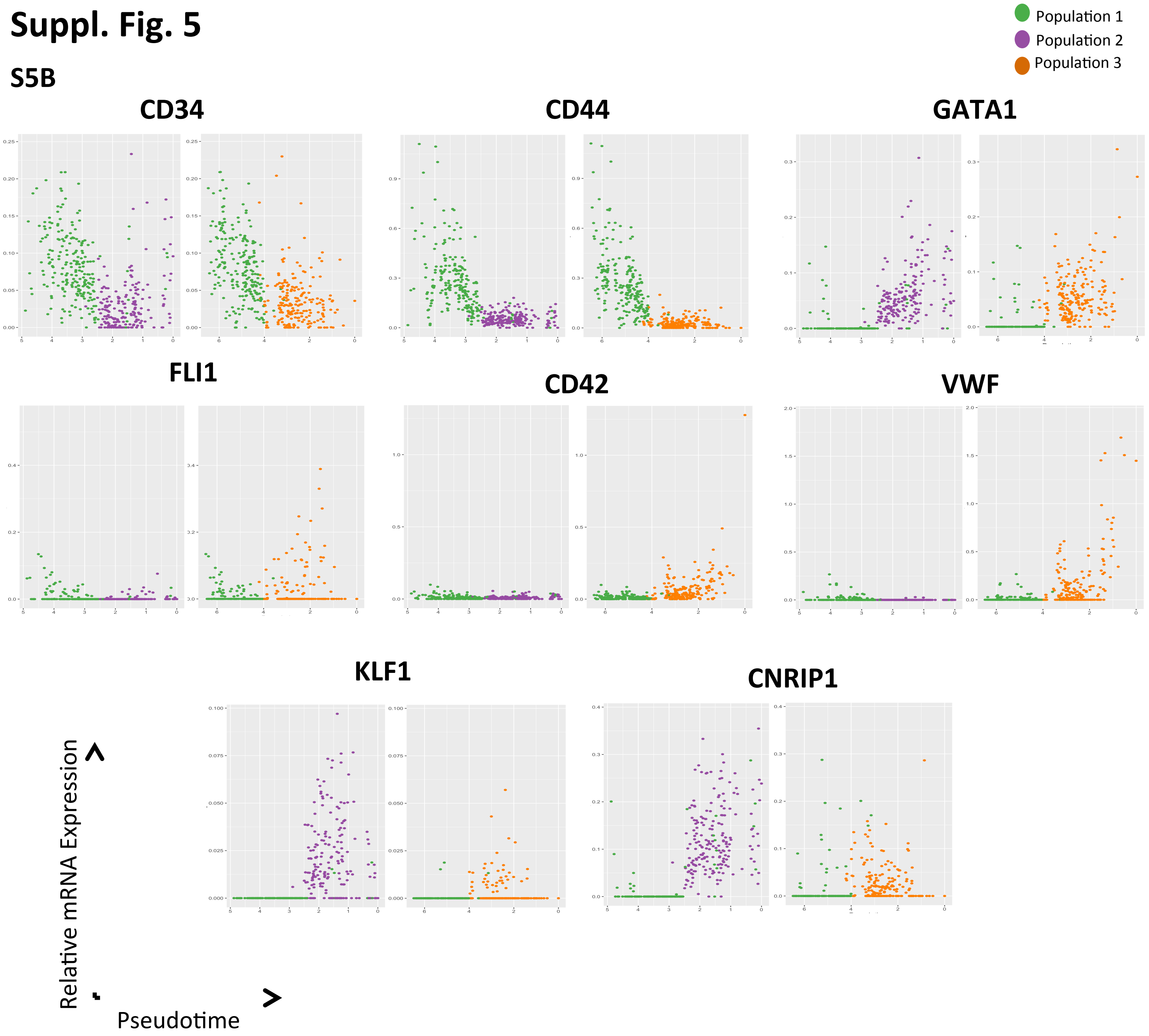
Population 1
Population 2
Population 3
S5B
CD34
CD44
GATA1
FLI1
CD42
VWF
KLF1
CNRIP1
Relative mRNA Expression
Pseudotime
